# RrMYB2 Regulates Drought Stress via RrJMJ12‐Dependent Epigenetic Modification in *Rosa rugosa*

**DOI:** 10.1111/pbi.70432

**Published:** 2025-10-28

**Authors:** Mengjuan Bai, Yating Yang, Yunfeng Gao, Mengmeng Xu, Qianxiang Zhang, Shuo Liu, Jun Lu, Jianwen Wang, Changquan Wang, Liguo Feng

**Affiliations:** ^1^ College of Horticulture and Landscape Architecture Yangzhou University Yangzhou China; ^2^ College of Horticulture Nanjing Agricultural University Nanjing China

**Keywords:** drought resistance, H3K27me3 modification, *R. rugosa*, *RrJMJ12*, *RrMYB2*

## Abstract

*Rosa rugosa* ( *R. rugosa* ), an economically important crop valued for its fragrance and medicinal properties, is highly susceptible to drought stress under open‐field cultivation. However, the molecular mechanisms underlying its drought response remain largely unexplored. Here, we identified RrMYB2, a transcription factor that positively regulates drought tolerance in *R. rugosa* . Overexpressing *RrMYB2* in *Arabidopsis thaliana* and *R. rugosa* enhanced drought tolerance. Virus‐induced silencing of *RrMYB2* plants exhibited impaired stomatal closure and reduced drought resistance. RNA‐seq of silenced plants revealed that *protein phosphatases type 2C* (*PP2C*) genes, including *RrHAB1*, *RrHAB2* and *RrABI1*, were significantly up‐regulated. Biochemical analyses demonstrated that the negative regulation of *PP2Cs* by RrMYB2 was dependent on its interaction protein RrJMJ12, an H3K27me3 histone demethylase and genetic evidence indicated that *RrJMJ12* acts downstream of *RrMYB2* to negatively regulate drought resistance. Moreover, the RrMYB2‐RrJMJ12 protein complex reduced RrJMJ12 binding to the CTCTGYTY motifs in the promoters of *PP2C* genes, with this inhibitory effect further enhanced under drought conditions. Collectively, our findings reveal a novel molecular mechanism in which a transcription factor cooperates with an epigenetic modifier to regulate drought tolerance in *R. rugosa* .

## 1 Introduction

Drought stress is a major abiotic factor limiting plant growth and crop productivity, leading to substantial economic losses worldwide. It induces a series of physiological and morphological changes in plants, manifested as leaf wilting, photosynthetic inhibition, growth suppression and ultimately plant death under severe conditions (Fang et al. 2015; Gupta et al. 2020). To withstand drought stress, plants have evolved complex physiological and molecular regulatory networks that optimise water use efficiency and maintain cellular homeostasis. Under drought stress, functional genes mediate diverse physiological responses, such as the accumulation of osmotic regulators, activation of antioxidant enzyme activity, detoxification of toxic metabolites and modification of cellular structure (Sahay et al. 2019; Singh et al. 2021). Concurrently, regulatory genes activate stress‐responsive signalling pathways by encoding signal transduction components or transcription factors, particularly those involved in the abscisic acid (ABA) signalling pathway, which serves as the core pathway in drought stress responses (Zhu 2016; You et al. 2023; Lozano‐Juste et al. 2023; Xiong et al. 2025). These molecular and physiological adaptations collectively mitigate the adverse effects of drought stress and enhance plant resilience.

Transcription factors play crucial roles in converting the stress‐induced signals into cellular responses. These include MYB, ERF, bZIP and WRKY proteins (Singh and Laxmi 2015). The MYB family constitutes one of the largest transcription factor classes in plants and is divided into four subgroups: 1R‐, R2R3‐, 3R‐ and 4R‐MYB based on the number of adjacent MYB domain repeats (Stracke et al. 2001; Dubos et al. 2010). Notably, most drought‐responsive MYB transcription factors belong to the R2R3‐MYB subgroup, including genes such as *AtMYB2*, *AtMYB15*, *AtMYB37* and *AtMYB44*, which confer enhanced drought tolerance when overexpressed in transgenic plants (Abe et al. 2003; Ding et al. 2009; Yu et al. 2016; Jung et al. 2008). In addition, MYB transcription factors regulate drought responses through the ABA pathway while interacting with other stress‐responsive regulatory networks. For instance, *AtMYB96* integrates ABA and auxin signalling to modulate drought responses (Seo et al. 2009). The wheat MYB transcription factor TaPIMP1 enhances both disease resistance and drought tolerance by synergistically regulating stress‐responsive genes in the ABA and salicylic acid (SA) signalling pathways (Zhang et al. 2012). These findings highlight the multifaceted and interconnected regulatory roles of MYB transcription factors in plant stress adaptation.

Accumulating evidence indicates that epigenetic modifications, including DNA methylation, histone methylation, acetylation and phosphorylation, critically regulate the expression of stress‐responsive genes (Chinnusamy and Zhu 2009; Kim et al. 2012; Li et al. 2023; Zhao et al. 2024; Gao et al. 2025). For instance, histone acetyltransferase *gcn5* mutants show enhanced heat stress tolerance due to altered H3K14 and H3K9 acetylation levels of *HSFA3* and *UVH6* genes (Hu et al. 2015). In rice, drought stress alters transcription of the histone acetyltransferase gene *OsHATs* and the acetylation levels of lysine residues on histones H3 and H4 (Fang et al. 2014). Similarly, in *Arabidopsis*, drought‐induced expression of stress‐responsive genes is associated with increased H3K4 trimethylation and H3K9 acetylation (Kim et al. 2008). In addition, histone modifications also interact with hormone signalling under abiotic stress. In *Arabidopsis*, the histone methyltransferase ATX1 promotes H3K4me3 accumulation at the promoter region of *NCED3*, a key gene in the ABA synthesis pathway, thereby promoting its expression under drought stress (Ding et al. 2011). In rice, different histone deacetylase family members exhibit distinct expression profiles in response to abiotic stressors, such as cold, osmotic and salt stress, as well as to hormone signalling pathways, including ABA, jasmonic acid (JA) and SA (Fu et al. 2007).

*R. rugosa* is an economically important crop valued for its fragrant flowers and medicinal properties. However, the open‐field cultivation renders it particularly vulnerable to drought stress, a challenge exacerbated by climate change. Despite its agricultural significance, the molecular mechanisms underlying drought responses in *R. rugosa* remain poorly understood. In this study, we identified the drought and ABA‐response gene *RrMYB2* from *R. rugosa* and demonstrated that it positively regulates drought tolerance. RrMYB2 indirectly represses the ABA signalling negative regulators *PP2C* genes, including *RrHAB1*, *RrHAB2* and *RrABI1*, via the H3K27me3 histone demethylase RrJMJ12. We indicated that *RrJMJ12* acts downstream of *RrMYB2* to negatively regulate drought resistance. The RrMYB2‐RrJMJ12 protein complex reduced the binding of RrJMJ12 to the CTCTGYTY motifs in the promoters of *PP2C* genes, thereby reducing their expression. Together, our findings provide a novel molecular mechanism by which transcription factors cooperate with epigenetic modifiers to regulate drought resistance in *R. rugosa* .

## 2 Materials and Methods

### 2.1 Plant Materials and Growth Conditions

The plant materials used in this study included *R. rugosa* ‘Zizhi’, *Rosa chinensis* ‘Old Blush’, *Arabidopsis thaliana* and *N. benthamiana*. All plants were cultivated in a greenhouse under controlled conditions (24°C, 16 h light/8 h dark).

Tissue culture seedlings of *R. rugosa* ‘Zizhi’ and ‘Old Blush’ were used for transient transformation experiments. Propagation and subculture of these tissue culture seedlings followed previously described methods (Lu et al. 2020).

### 2.2 Drought and ABA Stress Treatments

For gene expression analysis, *R. rugosa* ‘Zizhi’ plants were treated with 20% (w/v) PEG6000 and 100 μM ABA. Leaf samples were collected at specified time points for further analysis.

For transgenic assays mediated by *Agrobacterium rhizogenes* ( *A. rhizogenes* ), six‐week‐old plants were used for the natural drought. When leaf wilting was observed, chlorophyll fluorescence was measured. Chlorophyll fluorescence images and Y(II) content were recorded using the PlantView230F system (Biolight Biotechnology, Guangzhou, China). Root samples were collected for RT‐qPCR analysis and physiological parameter measurements. The physiological indicators of hydrogen peroxide (H_2_O_2_), superoxide anion (O_2_^−^), superoxide dismutase (SOD) and catalase (CAT) were quantified using assay kits (Jiancheng, Nanjing, China). For virus‐induced gene silencing (VIGS), leaf relative water content (RWC) was determined following previously described methods (Gaxiola et al. 2001).

For *Arabidopsis* seed germination assays, surface‐sterilised seeds were plated on 1/2MS medium plates (pH = 5.8) with or without ABA at varying concentrations. To synchronise germination, plates were stratified at 4°C for 3 days and then transferred to growth chambers maintained at 22°C under long‐day conditions (16 h light/8 h dark cycle).

For the drought treatments in *Arabidopsis*, 4‐week‐old plants were subjected to detached‐leaf water loss rate assays and natural drought stress. Leaf samples were collected for semi‐quantitative RT‐PCR analysis.

### 2.3 Phylogenetic Analysis

The sequence data for MYB S20 subgroup proteins in *Arabidopsis* were retrieved from the TAIR database (https://www.arabidopsis.org/). Protein sequences from *Arabidopsis* were used as BLAST queries to identify homologues in the *R. rugosa* protein database. The retrieved *R. rugosa* MYB S20 subgroup protein sequences, together with their *Arabidopsis* counterparts, were used to construct a phylogenetic tree using MEGA version 7.0.

### 2.4 Vector Construction

The coding sequences (CDSs) of *RrMYB2* and *RrJMJ12* were cloned into the pENTR D‐TOPO entry vector (Invitrogen, Carlsbad, CA, USA) and were then recombined via LR reaction into the pFAST‐R05 vectors containing a GFP tag to construct the overexpression vectors (35S:RrMYB2 and 35S:RrJMJ12), respectively. To generate RNA interference (RNAi) constructs, approximately 300 bp gene‐specific fragments of *RrMYB2* and *RrJMJ12* were cloned into the pENTR D‐TOPO entry vector and were then recombined via LR reaction into the pFAST‐R03 vector, yielding RrMYB2‐RNAi and RrJMJ12‐RNAi constructs, respectively. Additionally, the same fragments were inserted into the pTRV2 vector to generate VIGS constructs, TRV‐RrMYB2 and TRV‐RrJMJ12, respectively.

For electrophoretic mobility shift assay (EMSA), the C‐terminal region of *RrJMJ12* (1375–1497 aa) and its CDS were inserted into the pGEX4T‐1 and pET32a vectors to generate RrJMJ12‐C‐GST and RrJMJ12‐HIS fusion proteins, respectively. The CDS of *RrMYB2* was inserted into the pGEX4T‐1 vector to generate the RrMYB2‐GST fusion protein.

For transcriptional activation assays, the full‐length N‐terminal (1–160 aa), and C‐terminal (161–305 aa) regions of *RrMYB2* were cloned into the pGBKT7 vector to generate the RrMYB2 full‐length, RrMYB2‐N and RrMYB2‐C constructs.

For transient LUC activity and yeast one‐hybrid (Y1H) assays, the promoter sequences of *RrHAB1*, *RrHAB2* and *RrABI1* were inserted into pBGWL7 and pHIS2 vectors to generate the *pRrHAB1*:LUC, *pRrHAB2*:LUC and *pRrABI1*:LUC plasmids or *pRrHAB1*‐pHIS, *pRrHAB2*‐pHIS and *pRrABI1*‐pHIS plasmids.

### 2.5 Subcellular Localisation and Transcriptional Activation Assays

For subcellular localisation, *Agrobacterium tumefaciens* strain GV3101 carrying the 35S:RrMYB2 construct was used to transiently express RrMYB2‐GFP fusion protein in *N. benthamiana* leaves. Following agroinfiltration, the plants were incubated overnight in the dark at 25°C. Green fluorescence signals were observed within 48–72 h post‐infiltration using a confocal laser scanning microscope (LSM 880NLO, Carl Zeiss). GFP fluorescence was excited at 488 nm and detected at 500–535 nm.

For transcriptional activation, yeast strain Y2H was transformed with RrMYB2 full‐length RrMYB2‐N and RrMYB2‐C vectors. Positive colonies were selected on SD medium lacking tryptophan and leucine (SD/−Trp/−Leu) and subsequently transferred to SD medium lacking tryptophan, leucine, histidine and adenine (SD/−Trp/−Leu/−His/−Ade) to assess growth.

### 2.6 RNA Extraction and RT‐qPCR

Total RNA was extracted using the FastPure Plant Total RNA Isolation Kit (Polysaccharides & Polyphenolics–Rich) (Vazyme, Nanjing, China) following the manufacturer's instructions. First‐strand cDNA was synthesised using the HiScript III RT SuperMix for qPCR (+gDNA wiper) (Vazyme). Fluorescent quantitative PCR primers were designed based on the cDNA sequences and standard primer design principles. RT‐qPCR was performed using the CFX Connect Real‐Time PCR Detection System (Bio‐Rad, USA) with SYBR qPCR Master Mix (Vazyme) and gene‐specific primers (Table S1). Gene expression levels were analysed using the 2^−ΔΔCt^ method (Livak and Schmittgen 2001), with *Rr5.8 s* and *RrGAPDH* serving as the internal reference genes (Luo et al. 2018; Bai et al. 2021).

### 2.7 Transgenic Assays Mediated by *A. rhizogenes*

35S:RrMYB2, RrMYB2‐RNAi, 35S:RrJMJ12 and RrJMJ12‐RNAi (with 35S:Empty and Empty‐RNAi as controls) plasmids were introduced into *A. rhizogenes* K599 to obtain positive *Agrobacterium*. *R. rugosa* cuttings (lower end of morphology) were then immersed in the bacterial suspension and subsequently planted in a greenhouse under controlled conditions. Hairy roots emerged approximately 3 weeks post‐inoculation and were used for subsequent assays.

### 2.8 VIGS

VIGS was performed using the TRV system with the engineered vectors pTRV1 and pTRV2 (Liu et al. 2002). Briefly, *A. tumefaciens* cultures carrying pTRV1 and pTRV2, as well as the recombinant constructs TRV‐RrMYB2 or TRV‐RrJMJ12, were mixed at a 1:1 (v/v) ratio and adjusted to an OD_600_ of 1.0. Rose plantlets were submerged in the infiltration buffer and subjected to vacuum infiltration to ensure efficient gene delivery. Following infiltration, the plantlets were briefly rinsed and transferred to the growth substrate for subsequent experiments.

### 2.9 RNA Sequencing (RNA‐Seq)

Total RNAs of three independent TRV and TRV‐RrMYB2 plants were extracted for cDNA library construction. RNA‐seq of cDNA libraries was performed on the Illumina NovaSeq 6000 platform. Read counts per gene were calculated by mapping to the *Rosa chinensis* ‘Old Blush’ genome (Raymond et al. 2018). Differentially expressed genes (DEGs) were identified by the threshold of absolute value of log_2_ (fold change) ≥ 1 and FDR < 0.05. Genes were annotated to the Kyoto Encyclopedia of Genes and Genomes (KEGG) database by eggNOG (http://eggnog‐mapper.embl.de/) and enriched by analysing using the hypergeometric test.

### 2.10 Stomatal Observation Assays

Stomatal apertures on the adaxial surfaces of rose leaves were analysed using the nail polish imprinting method (Wu and Zhao 2017). Samples were observed with a ZEISS Axio Scope.A1 microscope (Carl Zeiss). Ten stomata were analysed per sample, and the width‐length ratio was measured using ImageJ (https://imagej.net/ij/).

### 2.11 Yeast One‐Hybrid (Y1H) Assays

*pRrHAB1*‐pHIS, *pRrHAB2*‐pHIS and *pRrABI1*‐pHIS constructs were first tested individually in yeast strain Y187 to determine the optimal 3‐amino‐1,2,4‐triazole (3‐AT) concentration to suppress self‐activation. Then, *pRrHAB1*‐pHIS, *pRrHAB2*‐pHIS and *pRrABI1*‐pHIS vectors were co‐transformed into the yeast cell Y187 with RrMYB2‐pGADT7, respectively. Positive colonies were selected on SD/−Trp/−Leu/−His medium supplemented with the optimised concentration of 3‐AT.

### 2.12 Electrophoretic Mobility Shift Assay (EMSA) and Pull‐Down Assay

Recombinant proteins RrJMJ12‐C‐GST, RrJMJ12‐HIS and RrMYB2‐GST were expressed following isopropyl β‐D‐1‐thiogalactoside (IPTG) induction. Biotin‐labelled probes and competitor probes were designed based on motif positions (Table S1). EMSA was performed using the EMSA/Gel‐Shift Kit (Beyotime Biotechnology, Shanghai, China). Proteins were incubated with probes in binding buffer for 30 min at 24°C, separated on acrylamide gels and visualised on a Tanon 4600SF system (Tanon, Shanghai, China).

For pull‐down assays, recombinant RrJMJ12‐HIS and RrMYB2‐GST fusion proteins were mixed in equal volumes, incubated and purified on a GST column using a GST‐Tagged Protein Purification Kit (CWBIO, Beijing, China). Finally, a western blot assay was performed using HIS or GST antibodies.

### 2.13 Transient Transformation Analysis in Rose Seedlings

Transient transformations were conducted as previously described (Lu et al. 2017). Briefly, sub‐cultured rose seedlings were submerged in about 50 mL of *Agrobacterium* cell suspension carrying different constructs and vacuum‐infiltrated at 0.5 MPa for 5 min. Seedlings were washed with distilled water and placed on water‐soaked filter paper. LUC activity was detected within 48–72 h using a CCD camera (Tanon 4600SF, Tanon, Shanghai, China).

### 2.14 Bimolecular Fluorescence Complementation (BiFC) Assays

The CDSs of *RrMYB2* and *RrJMJ12* without stop codons were individually inserted into the 2YN (YFP‐n) or 2YC (YFP‐c) vectors by homologous recombination using the Hieff Clone Plus One Step Cloning Kit (YEASEN, Nanjing, China). The resulting constructs were then introduced into *Agrobacterium* GV3101. *Agrobacterium* cultures, each harbouring one plasmid, were mixed and co‐infiltrated into the leaves of genetically modified *N. benthamiana* accumulating nucleus‐localised red fluorescent protein (RFP) as a positive control. YFP and RFP fluorescence signals were observed 48–72 h post‐infiltration using a confocal microscope (Zeiss LSM800, Carl Zeiss, Oberkochen, Germany).

### 2.15 Split‐LUC Complementation Assays

The CDSs of *RrMYB2* and *RrJMJ12* were cloned into the pENTR D‐TOPO entry vector and were then recombined via LR reaction into the binary vector pMK7‐cL‐WG2 or pMK7‐nL‐WG2 to produce RrMYB2:LUC‐N or RrJMJ12:LUC‐C, respectively. Constructs were introduced into *Agrobacterium* GV3101 and co‐infiltrated into *N. benthamiana* leaves. LUC activity was measured 48–72 h post‐infiltration using a CCD camera (Andor Technology, Belfast, UK). Primers are listed in Table S1.

### 2.16 Chromatin Immunoprecipitation PCR (ChIP‐PCR)

To test H3K27me3 modification levels on the *RrHAB1*, *RrHAB2* and *RrABI1* genes, ChIP assays were performed using rose seedlings harbouring RrJMJ12‐RNAi or 35S:RrJMJ12, with H3K27me3 antibodies. For the ChIP‐qPCR assay that detects the binding of RrJMJ12 to the CTCTGYTY motifs under normal and drought conditions, the rose seedlings were subjected to normal and drought treatments. When drought‐treated plants exhibited slight wilting, leaves were collected for transient expression and ChIP‐qPCR with GFP antibodies. All ChIP assays followed previously described methods (Bai et al. 2021), and the primers are listed in Table S1.

## 3 Results

### 3.1 Characterisation of RrMYB2

**FIGURE 1:**
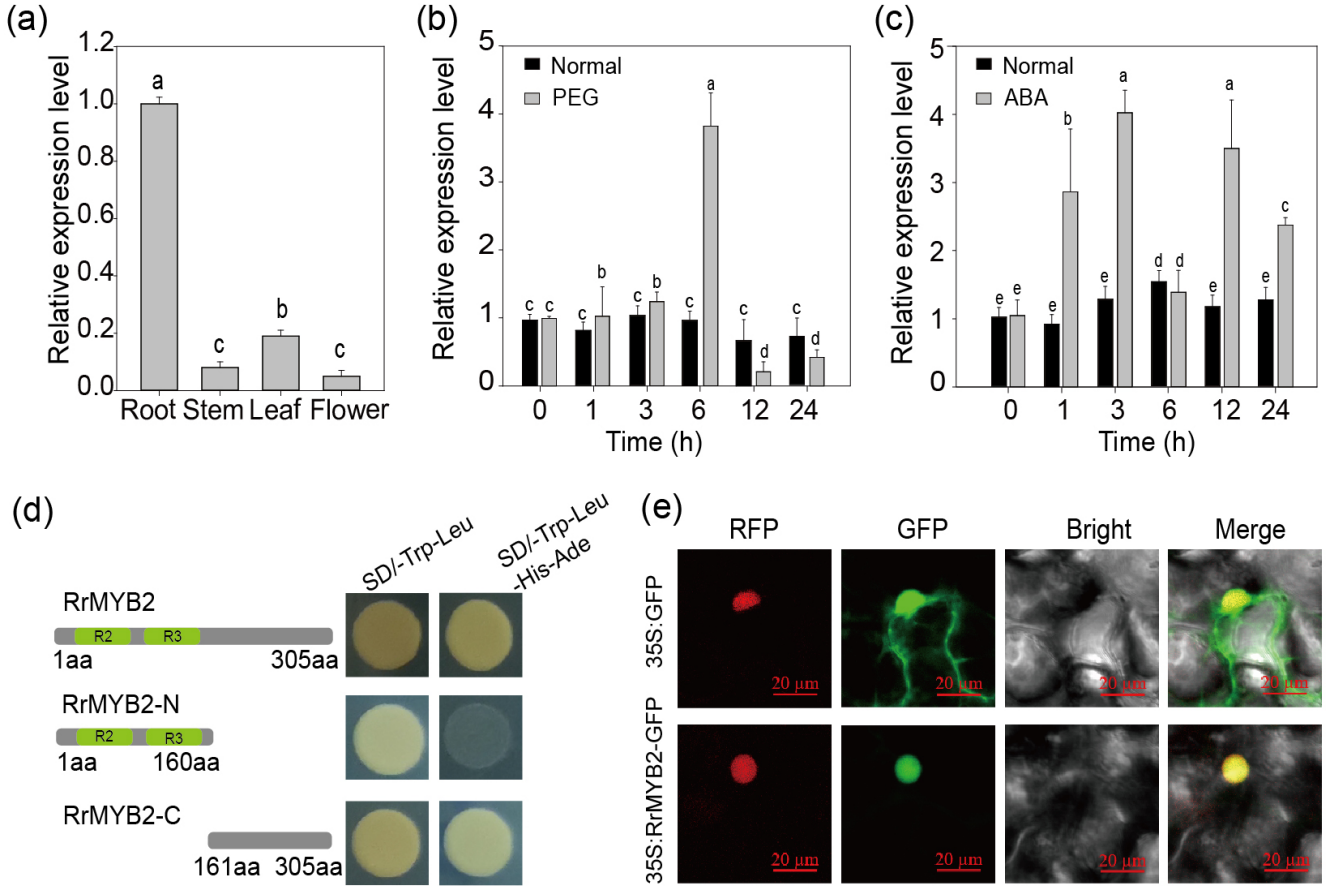
Expression analysis and protein characteristics of RrMYB2. (a) Relative expression levels of *RrMYB2* in different tissues of *R. rugosa* . (b, c) Expression analysis of *RrMYB2* in response to 20% PEG (b) and ABA (c) treatments. Values represent the means ±SD from three replicates. Different letters above the bars indicate significant differences determined by one‐way ANOVA analysis followed by Duncan's test (*p* < 0.05). *Rr5.8 s* as a reference gene. (d) Transcriptional activation assay of RrMYB2 full length, RrMYB2‐N (1–160 aa) and RrMYB2‐C (161–305 aa) in yeast system. (e) Subcellular localisation of RrMYB2 proteins in *N. benthamiana* epidermal cells carrying a nuclear localised RFP signal. Bars = 20 μm.

In *Arabidopsis*, R2R3‐MYB proteins are classified into 25 subgroups (S1–S25), with several subgroups (S1, S2, S11, S18, S20 and S22) being involved in regulating biotic and abiotic stress responses (Agarwal et al. 2006; Bolton et al. 2006; Seo et al. 2009; Dubos et al. 2010; Froidure et al. 2010). *AtMYB2*, a member of the S20 subgroup, plays a key role in drought stress tolerance (Abe et al. 1997, 2003). To identify the *RrMYB2* gene in *R. rugosa* , we screened the genome database of *R. rugosa* ‘Zizhi’ and identified eight S20 subgroup genes (Figure S1a). Phylogenetic analysis revealed that *AtMYB2* shared the highest sequence similarity with Rru03G033970, which encodes a 305‐amino‐acid (aa) protein containing a highly conserved R2R3‐MYB domain at its N‐terminus (Figure S1b). Tissue‐specific expression analysis showed that *RrMYB2* was highly expressed in roots and leaves, with the lowest expression observed in flowers (Figure 1a).

Subsequently, to determine whether *RrMYB2* participates in drought stress and ABA responses, we examined its expression under PEG‐induced drought stress and ABA treatment. The results showed that *RrMYB2* was significantly up‐regulated following both treatments (Figure 1b,c), suggesting a role in drought response regulation. To further characterise RrMYB2, we assessed its transcriptional activation activity in yeast. The results demonstrated that RrMYB2 exhibited strong transcriptional activation activity, with the activation domain located in the 161 to 305 aa region (Figure 1d). Moreover, subcellular localisation analysis in *N. benthamiana* leaves confirmed that RrMYB2 is nucleus‐localised (Figure 1e). Collectively, these findings indicate that *RrMYB2* is a typical R2R3‐MYB gene that plays a crucial role in drought and ABA responses in *R. rugosa* .

### 3.2 *RrMYB2* Positively Regulates Drought Tolerance in *Arabidopsis* and *R. rugosa*

To characterise the function of *RrMYB2*, we heterologously overexpressed *RrMYB2* in *Arabidopsis*. Transgenic lines (RrMYB2‐OE) were confirmed by semi‐quantitative RT‐PCR (Figure S2b). Three‐week‐old WT and RrMYB2‐OE seedlings were subjected to natural drought stress (Figure S2a). After 10 days, all RrMYB2‐OE plants survived, whereas none of the WT plants survived (Figure S2c). To further evaluate drought tolerance, we analysed water loss rates from detached leaves of WT and RrMYB2‐OE plants. The results showed that WT leaves lost water more rapidly than those of RrMYB2‐OE lines (Figure S2d). Seed germination assays further revealed that, whereas germination was unaffected in the absence of ABA, RrMYB2‐OE seeds were more sensitive to ABA at 0.7–5 μM, exhibiting significantly reduced germination compared with WT (Figure S2e). These results demonstrate that *RrMYB2* functions as a transcriptional activator in the ABA signalling pathway, positively regulating drought tolerance in *Arabidopsis*.

**FIGURE 2:**
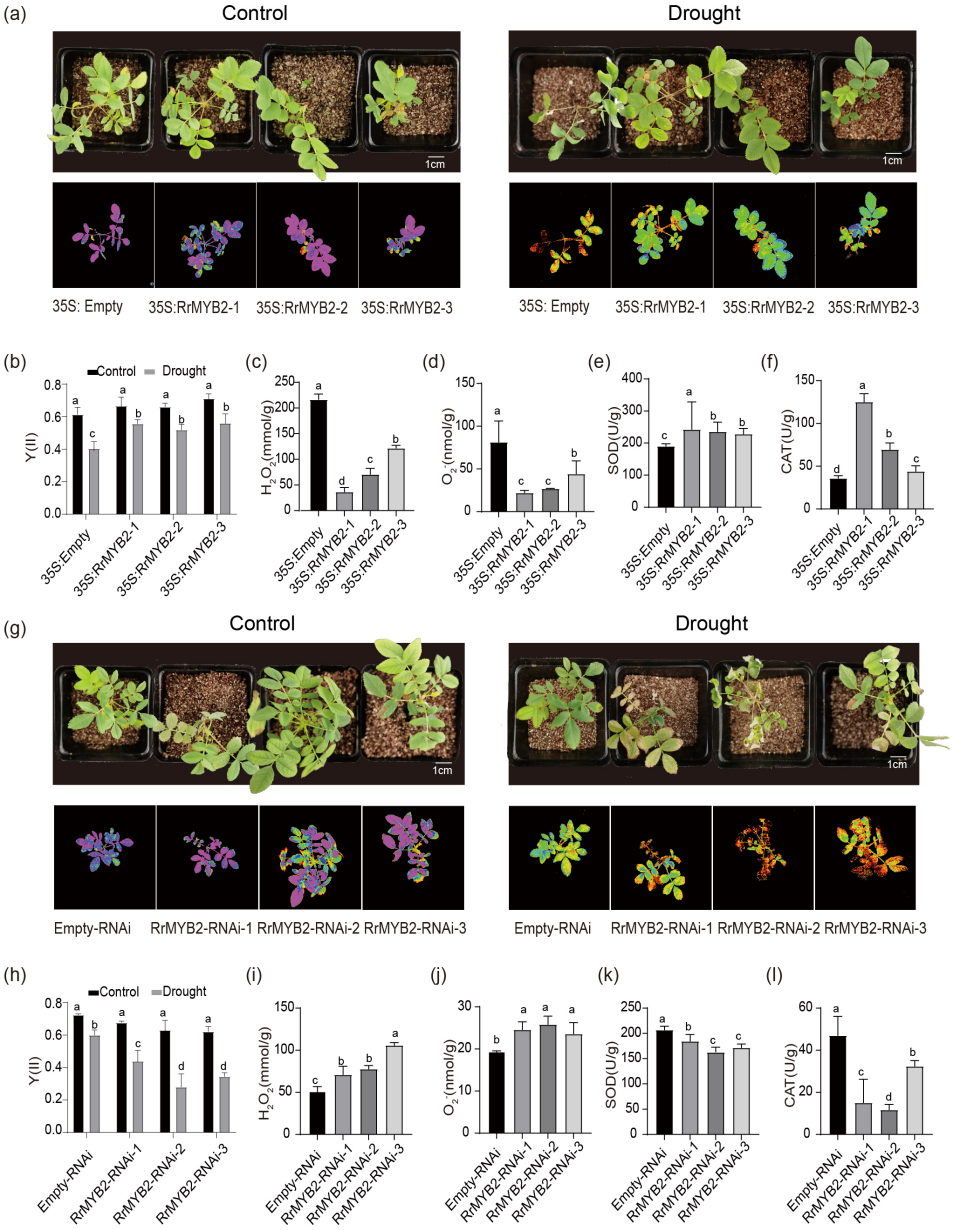
*RrMYB2* enhances drought tolerance in *R. rugosa* . (a, g) Growth phenotypes and corresponding chlorophyll fluorescence images of control (35S:Empty and Empty‐RNAi) and transgenic (35S:RrMYB2 and RrMYB2‐RNAi) *R. rugosa* plants before and after drought treatment. Bars = 1 cm. (b, h) Effective quantum yield (Y) of CK (35S:Empty and Empty‐RNAi) and transgenic (35S:RrMYB2 and RrMYB2‐RNAi) *R. rugosa* plants before and after drought treatment. (c–f) Contents of H_2_O_2_ (c) and O_2_^−^ (d) and activities of SOD (e), and CAT (f) in 35S:Empty and 35S:RrMYB2 transgenic roots of *R. rugosa* under drought conditions. (i–l) Contents of H_2_O_2_ (i), O_2_^−^ (j) and activities of SOD (k), and CAT (l) in Empty‐RNAi and RrMYB2‐RNAi transgenic roots of *R. rugosa* under drought conditions. Values represent the means ±SD from three replicates. Different letters above the bars indicate significant differences determined by one‐way ANOVA analysis followed by Duncan's test (*p* < 0.05).

To clarify the functions of *RrMYB2* in *R. rugosa* , we generated composite plants with transgenic roots using *A. rhizogenes* . Overexpression (35S:RrMYB2) and interference (RrMYB2‐RNAi) plants were confirmed by RT‐qPCR analysis (Figure S3a,b). Under normal conditions, no significant phenotypic differences were observed among the transgenic lines. However, after drought treatment, phenotypic observations and chlorophyll fluorescence imaging revealed that 35S:RrMYB2 plants exhibited less damage compared with 35S:Empty controls (Figure 2a,b). Consistently, 35S:RrMYB2 plants exhibited significantly lower levels of ROS (O_2_^−^ and H_2_O_2_) and higher activities of antioxidant enzyme activity (SOD and CAT) than 35S:Empty controls (Figure 2c–f). Conversely, RrMYB2‐RNAi plants exhibited more severe damage, higher ROS accumulation and reduced activities of SOD and CAT compared to Empty‐RNAi controls (Figure 2g–l). These results demonstrate that *RrMYB2* may inhibit excessive ROS accumulation by promoting antioxidant enzyme activity, thereby enhancing drought resistance in *R. rugosa* .

### 3.3 Silencing *RrMYB2* Affects Stomatal Closure

**FIGURE 3:**
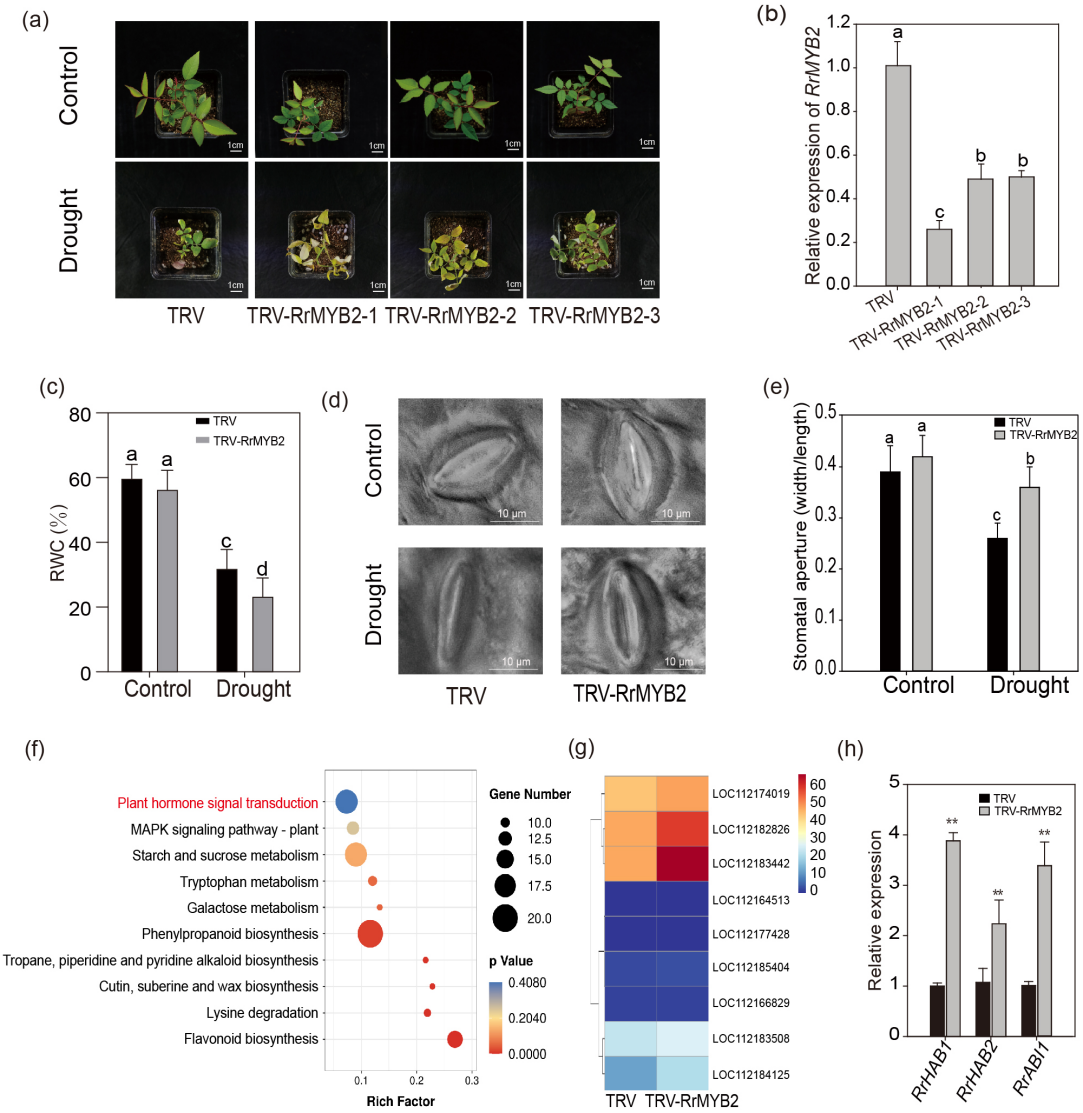
Silencing *RrMYB2* reduces drought tolerance in roses. (a) Phenotypes of TRV and TRV‐RrMYB2 plants before and after drought stress. Bars = 1 cm. (b) Expression levels of *RrMYB2* in TRV and TRV‐RrMYB2 rose plants. (c) RWC in the leaves of TRV and TRV‐RrMYB2 plants under control and drought stress conditions. (d, e) Stomatal morphology (d) and stomatal aperture size (e) of TRV and TRV‐RrMYB2 plants under control and drought stress conditions. Values represent the means ±SD. 10 stomata from three independent experiments. Bars = 10 μm. (f) KEGG enrichment analysis of DEGs in TRV and TRV‐RrMYB2 rose plants. (g) Heat map analysis of PP2Cs genes in TRV‐RrMYB2 compared with TRV. (h) Expression analysis of *RrHAB1*, *RrHAB2* and *RrABI1* in TRV and TRV‐RrMYB2 plants. *Rr5.8 s* was used as reference gene. Values represent the means ±SD from three replicates. Different letters above the bars indicate significant differences determined by one‐way ANOVA analysis followed by Duncan's test (*p* < 0.05). Asterisks indicate significant differences determined by Student's t tests (**p < 0.01).

To more deeply explore the role of RrMYB2 in rose drought stress, we silenced *RrMYB2* in rose using VIGS (Figure 3a). Silencing efficiency was confirmed by RT‐qPCR (Figure 3b). Under control conditions, no significant differences in RWC or stomatal aperture were observed between TRV and TRV‐RrMYB2 plants (Figure 3c–e). After drought, TRV‐RrMYB2 plants displayed severe wilting and leaf yellowing, accompanied by a significantly lower RWC than TRV plants. Stomatal apertures were also substantially larger in TRV‐RrMYB2 than in TRV plants (Figure 3a,c–e).

Next, we performed RNA‐seq to identify DEGs between TRV and TRV‐RrMYB2 plants. A total of 2310 DEGs were identified, including 800 up‐regulated and 1510 downregulated genes (Figure S4a). KEGG‐enrichment analysis of DEGs indicated significant enrichment in signalling pathways (‘plant hormone signal transduction’ and ‘MAPK signaling pathway‐plant’), metabolic pathways (‘Starch and sucrose metabolism’, ‘Tryptophan metabolism’ and ‘Galactose metabolism’) and biosynthesis pathways (‘Phenylpropanoid biosynthesis’, ‘Tropane, piperidine and pyridine alkaloid biosynthesis’, ‘Cutin, suberine and wax biosynthesis’ and ‘Flavonoid biosynthesis’) (Figure 3f and Table S2). Since *RrMYB2* was induced by ABA (Figure 1c), we analysed the DEGs in the plant hormone signalling pathway. Notably, the KEGG term K14497, encoding *protein phosphatase 2C* (*PP2C*) family proteins, was significantly enriched. Further heatmap analysis showed that four *PP2C* genes, LOC112183442 (*RrHAB1*), LOC112174019 (*RrHAB2*), LOC112184125 (*RrABI1*) and LOC112182826 (*RrPP2CA*), were significantly up‐regulated in TRV‐RrMYB2 plants (Figure 3g and Table S3). In *Arabidopsis*, the *hab1‐1 abi1‐2* double mutant exhibits strong ABA hypersensitivity, stomatal closing and drought resistance (Saez et al. 2004, 2006). Therefore, we performed RT‐qPCR analysis on *RrHAB1*, *RrHAB2* and *RrABI1*, which confirmed significant upregulation of all three *PP2C* genes in TRV‐RrMYB2 plants (Figure 3h). The consistent expression profiles of these *PP2C* genes were also verified in both 35S:RrMYB2 and RrMYB2‐RNAi plants (Figure S3c,d).

In addition, Gene Ontology (GO) enrichment analysis showed that the DEGs were enriched in biological processes related to ‘defense response’ and ‘response to stress’ (Figure S4b). Notably, the expression of nine *plant‐specific class III peroxidases* (*PERs*) genes was altered, implying that *RrMYB2* might regulate ROS levels by regulating these genes (Table S4). Furthermore, ten stress‐related genes were selected for PCR validation, including *RrLEA14*, *RrNCED3*, *RrCER1*, *RrFAR1*, *RrUGT71B6*, *RrFLS1*, *RrTT4*, *RrANS*, *RrF3H* and *RrPP2CA* (Figure S4c). The results demonstrated that all these genes were differentially regulated in both TRV and TRV‐RrMYB2 plants, which was consistent with the RNA‐seq results (Table S5).

### 3.4 RrJMJ12 Binds to the CTCTGYTY Motifs in the Promoters of *RrHAB1* , *RrHAB2* and *RrABI1*

MYB transcription factors regulate gene expression by binding to MYB‐binding elements in the promoters (Li, Han, et al. 2019). To investigate whether RrMYB2 exerts a similar regulatory function, we analysed *cis*‐elements in the promoters of *RrHAB1*, *RrHAB2* and *RrABI1* and identified MYB‐binding sites (Figure S5). However, Y1H assays demonstrated that RrMYB2 does not directly bind to the promoters of *RrHAB1*, *RrHAB2* and *RrABI1* (Figure S6a). Despite this, transient expression assays in rose seedlings showed that 35S:RrMYB2 significantly inhibited LUC activity driven by the *pRrHAB1*, *pRrHAB2* and *pRrABI1* promoters (Figure S6b–g). These results suggested that other factors may be required for *RrMYB2* to regulate the expression of *RrHAB1*, *RrHAB2* and *RrABI1* genes.

**FIGURE 4:**
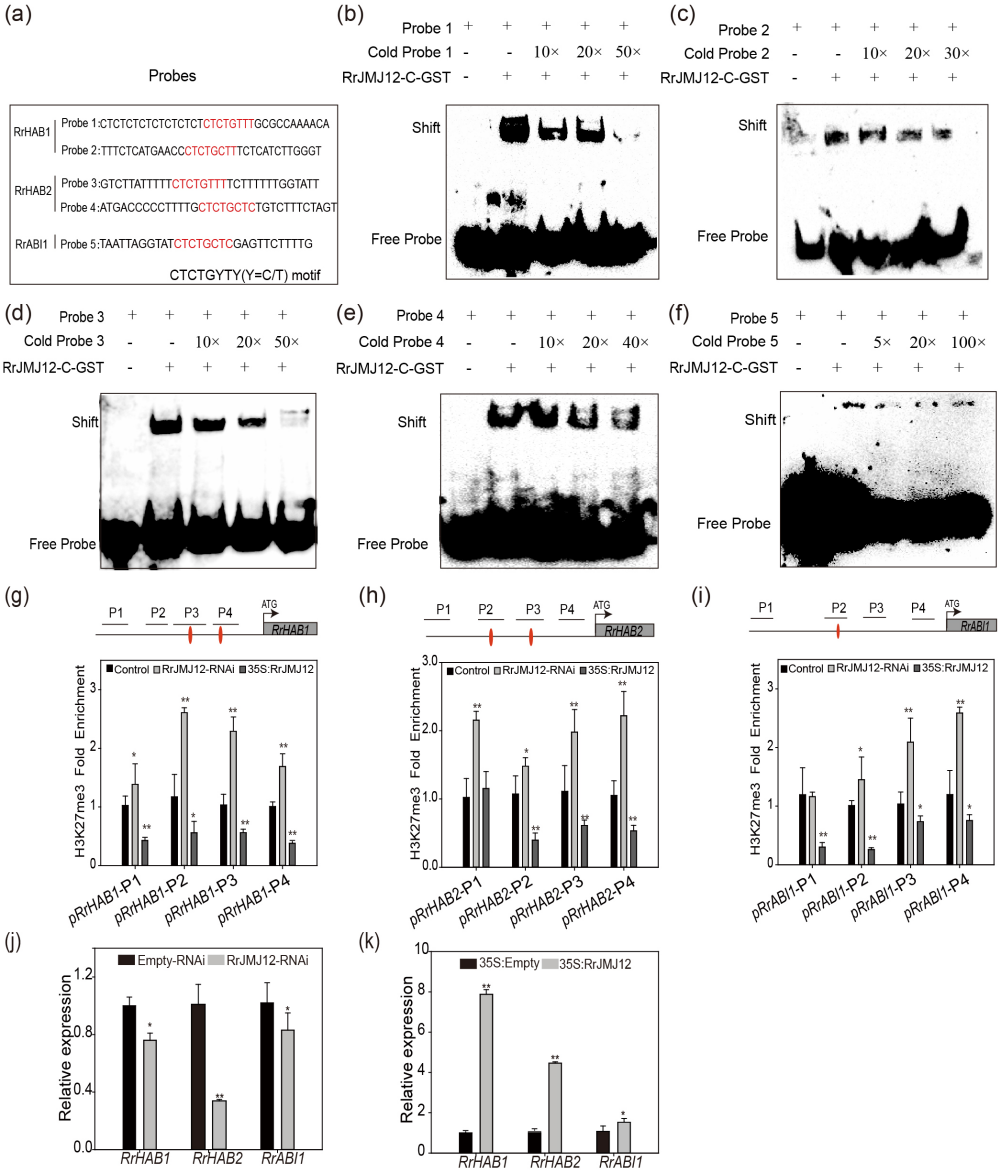
RrJMJ12 directly binds to the CTCTGYTY motifs. (a) Probe sequences containing the CTCTGYTY (Y=C/T) motif in the *RrHAB1*, *RrHAB2* and *RrABI1* promoters were designed. The CTCTGYTY (Y=C/T) motifs are highlighted in red. (b–f) EMSA verified RrJMJ12‐C protein binding to probes 1–5 in vitro. Biotin‐labelled probes were used, while unlabeled competitive probes (cold probes) served as controls. ‘+’ and ‘−’ indicate the presence and absence, respectively, of the indicated probe or protein. (g–i) Enrichment of H3K27me3 at different regions of the *RrHAB1*, *RrHAB2* or *RrABI1* promoters in *R. rugosa* transgenic seedlings. P1–P4 regions were selected for ChIP‐qPCR according to their locations. Red elliptic spots denoted as CTCTGYTY (Y=C/T) motif. (j, k) Expression levels of *RrHAB1*, *RrHAB2* and *RrABI1* in *R. rugosa* transgenic seedlings. Values represent the means ±SD from three replicates. Asterisks indicate significant differences determined by Student's *t* tests (*p < 0.05, **p < 0.01).

Many studies have shown that histone methylation modifications play crucial roles in plant abiotic stress responses (Chinnusamy and Zhu 2009). JMJ demethylases, including JMJ17, JMJ27, JMJ14 and JMJ12, modulate drought or heat responses by removing H3K4me3, H3K9me2 and H3K27me3 marks on target genes (Huang et al. 2019; Wang et al. 2021; Cui et al. 2021; Liu et al. 2019). In this study, *cis‐element* analysis identified conserved JMJ12 binding motifs (CTCTGYTY, Y = C/T) in the promoters of *RrHAB1*, *RrHAB2* and *RrABI1* (Cui et al. 2016) (Figure S5), which prompted us to verify whether RrJMJ12 is a potential co‐regulator required for RrMYB2. To prove this hypothesis, we first generated the RrJMJ12‐C‐GST fusion proteins, including the C‐terminal region (1375 aa–1497 aa) of RrJMJ12, and probes were designed at the position of the CTCTGYTY motifs (Figure 4a). EMSAs confirmed that RrJMJ12‐C specifically bound to the CTCTGYTY motifs in all three promoters (Figure 4b–f).

As RrJMJ12 is a histone demethylase that specifically demethylates H3K27me2/3 histone marks (Lu et al. 2011). We asked whether *RrHAB1*, *RrHAB2* and *RrABI1* expression is associated with H3K27me3 enrichment. Rose seedlings transiently expressing 35S:RrJMJ12 or RrJMJ12‐RNAi were subjected to ChIP‐qPCR using H3K27me3 antibodies. RT‐qPCR confirmed successful overexpression and silencing of *RrJMJ12* in the respective seedlings (Figure S7). Subsequently, we designed specific primers targeting the promoter regions of *RrHAB1*, *RrHAB2* and *RrABI1* and performed ChIP‐qPCR to assess H3K27me3 enrichment levels. ChIP‐qPCR showed significantly higher H3K27me3 enrichment at the promoters of *RrHAB1*, *RrHAB2* and *RrABI1* in RrJMJ12‐RNAi seedlings, whereas enrichment decreased in 35S:RrJMJ12 seedlings (Figure 4g–i). Correspondingly, the expression of these three genes decreased in RrJMJ12‐RNAi seedlings but increased in 35S:RrJMJ12 seedlings (Figure 4j,k). These results indicated that RrJMJ12 regulates the expression of *RrHAB1*, *RrHAB2* and *RrABI1* genes by modulating their H3K27me3 levels in *R. rugosa* .

### 3.5 RrMYB2 Represses the Expression of *RrHAB1* , *RrHAB2* and *RrABI1* in an RrJMJ12‐Dependent Manner

**FIGURE 5:**
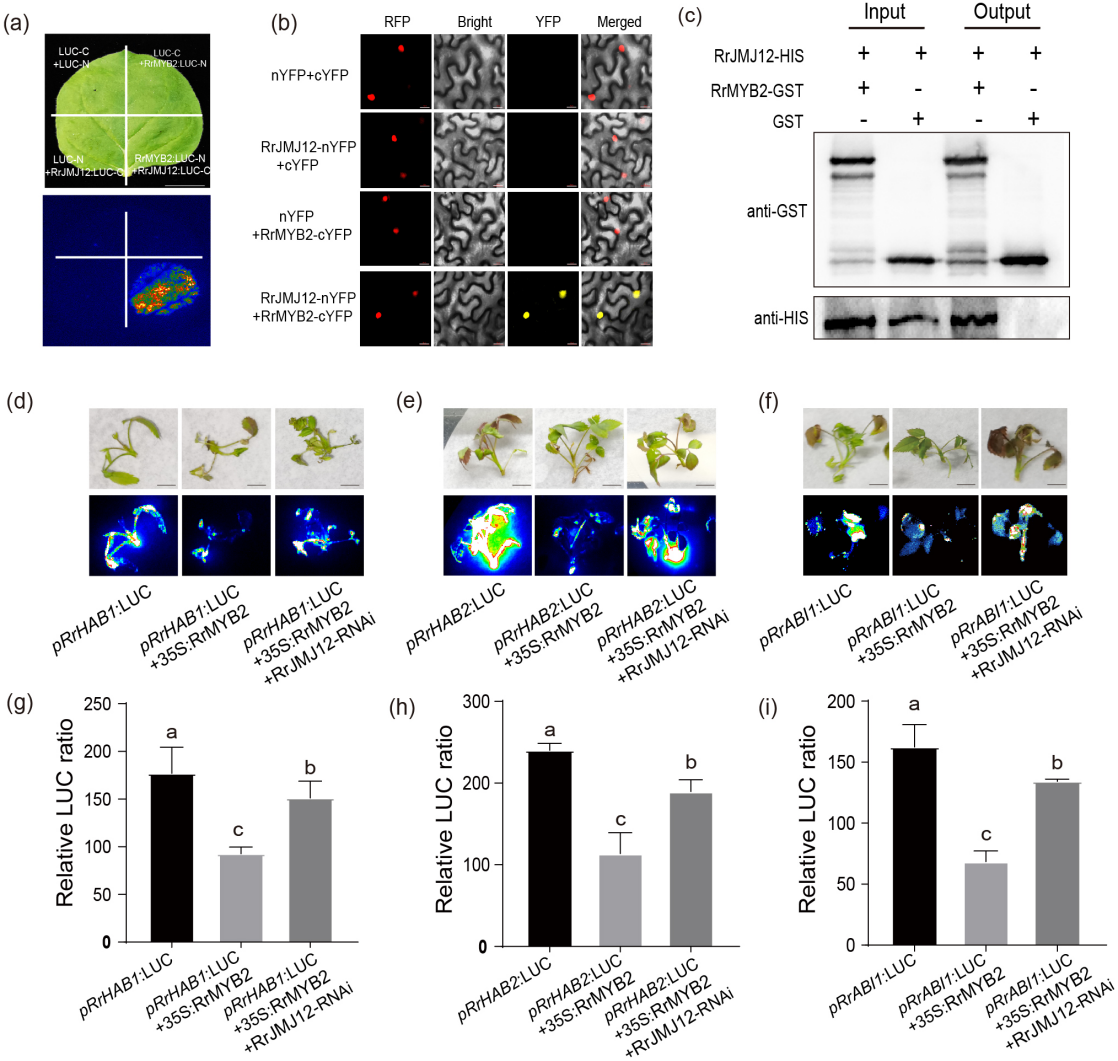
RrMYB2 inhibition of *RrHAB1*, *RrHAB2* and *RrABI1* gene expression depends on RrJMJ12. (a) Split‐LUC complementation assay verified the interaction between RrMYB2 and RrJMJ12 in leaves of *N. benthamiana*. Bar = 2 cm. (b) BiFC assays verified the interaction between RrMYB2 and RrJMJ12 in leaves of *N. benthamiana* carrying a nuclear localised RFP. Bars = 20 μm. (c) Interaction between RrMYB2 and RrJMJ12 in pull‐down assay. In vitro‐translated GST protein was used as a negative control. ‘Input’ refers to the protein mixtures before the experiment. ‘Output’ means the purified protein mixture. The ‘+’ indicates an existence, and the ‘−’ indicates a non‐existence. (d–f) Representative images of LUC transient expression assays displayed by bright field and dark field of rose seedlings of expressing *pRrHAB1*/*pRrHAB2*/*pRrABI1*:LUC either alone or together with 35S:RrMYB2 and RrJMJ12‐RNAi in a 1:1 or 1:1:1 ratio, respectively. Bars = 5 mm. (g–i) LUC activity in (d–f) were calculated using Andor Solis software. Means ± SE were shown from about 10 replicates. Different letters above the bars indicate significant differences determined by one‐way ANOVA analysis followed by Duncan's test (*p* < 0.05).

Our research results confirmed that both RrMYB2 and RrJMJ12 can regulate the expression of *RrHAB1*, *RrHAB2* and *RrABI1* (Figures 3, 4), which prompted us to investigate the interaction between RrMYB2 and RrJMJ12. Split‐LUC complementation, BiFC and pull‐down assays consistently confirmed their physical interaction (Figure 5a–c).

Next, to further validate our previous hypothesis, we performed transient transformation experiments in rose seedlings. Interestingly, the inhibitory effect of RrMYB2 on *RrHAB1*, *RrHAB2* and *RrABI1* expression was almost eliminated in *RrJMJ12*‐silenced seedlings (Figure 5d–i). These findings demonstrate that *RrMYB2* represses *PP2Cs* gene expression through an RrJMJ12‐dependent mechanism that requires both RrJMJ12 binding to CTCTGYTY motifs and its physical interaction with RrMYB2.

### 3.6 *RrJMJ12* Negatively Regulates Drought Tolerance in *R. rugosa*

**FIGURE 6:**
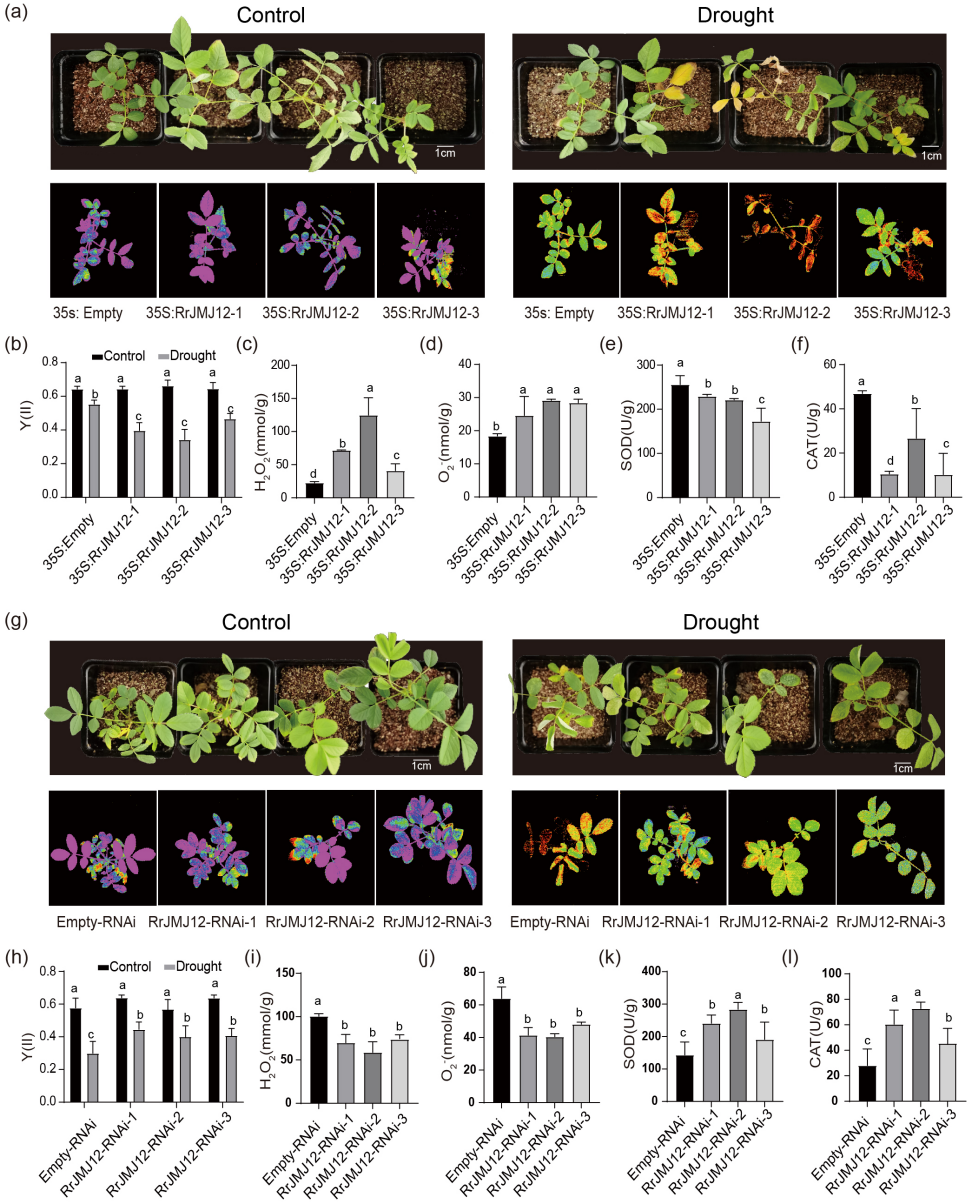
*RrJMJ12* negatively regulates drought tolerance in *R. rugosa* . (a, g) Growth phenotypes and corresponding chlorophyll fluorescence images of control (35S:Empty and Empty‐RNAi) and transgenic (35S:RrJMJ12 and RrJMJ12‐RNAi) *R. rugosa* plants before and after drought treatment. Bars = 1 cm. (b, h) Effective quantum yield (Y) of CK (35S:Empty and Empty‐RNAi) and transgenic (35S:RrJMJ12 and RrJMJ12‐RNAi) *R. rugosa* plants before and after drought treatment. (c–f) Contents of H_2_O_2_ (c) and O_2_^−^ (d) and activities of SOD (e), and CAT (f) in roots of 35S:Empty and 35S:RrJMJ12 transgenic *R. rugosa* plants under drought conditions. (i–l) Contents of H_2_O_2_ (i), O_2_^−^ (j) and activities of SOD (k), and CAT (l) in roots of Empty‐RNAi and RrJMJ12‐RNAi transgenic *R. rugosa* plants under drought conditions. Values represent the means ± SD from three replicates. Different letters above the bars indicate significant differences determined by one‐way ANOVA analysis followed by Duncan's test (*p* < 0.05).

To investigate the role of RrJMJ12 in drought stress, we also obtained *RrJMJ12* overexpression (35S:RrJMJ12) and interference (RrJMJ12‐RNAi) plants using *A. rhizogenes* ‐mediated transformation. Transgenic plants were confirmed by RT‐qPCR (Figure S8a,b). Following drought treatment, phenotypic observations and chlorophyll fluorescence imaging revealed that 35S:RrJMJ12 plants exhibited more severe damage compared with 35S:Empty controls (Figure 6a,b). Physiological indicators analysis showed that 35S:RrJMJ12 plants accumulated higher levels of H_2_O_2_ and O_2_^−^, while exhibiting lower SOD and CAT activities than 35S:Empty plants (Figure 6c–f). Conversely, RrJMJ12‐RNAi plants showed less damage, accumulated less H_2_O_2_ and O_2_^−^, and exhibited higher SOD and CAT activities compared with Empty‐RNAi plants (Figure 6g–l). *RrHAB1*, *RrHAB2* and *RrABI1* expression levels were significantly up‐regulated in 35S:RrJMJ12 plants and significantly down‐regulated in RrJMJ12‐RNAi plants (Figure S8c,d). These results indicate that *RrJMJ12* functions as a negative regulator of drought tolerance in *R. rugosa* .

**FIGURE 7:**
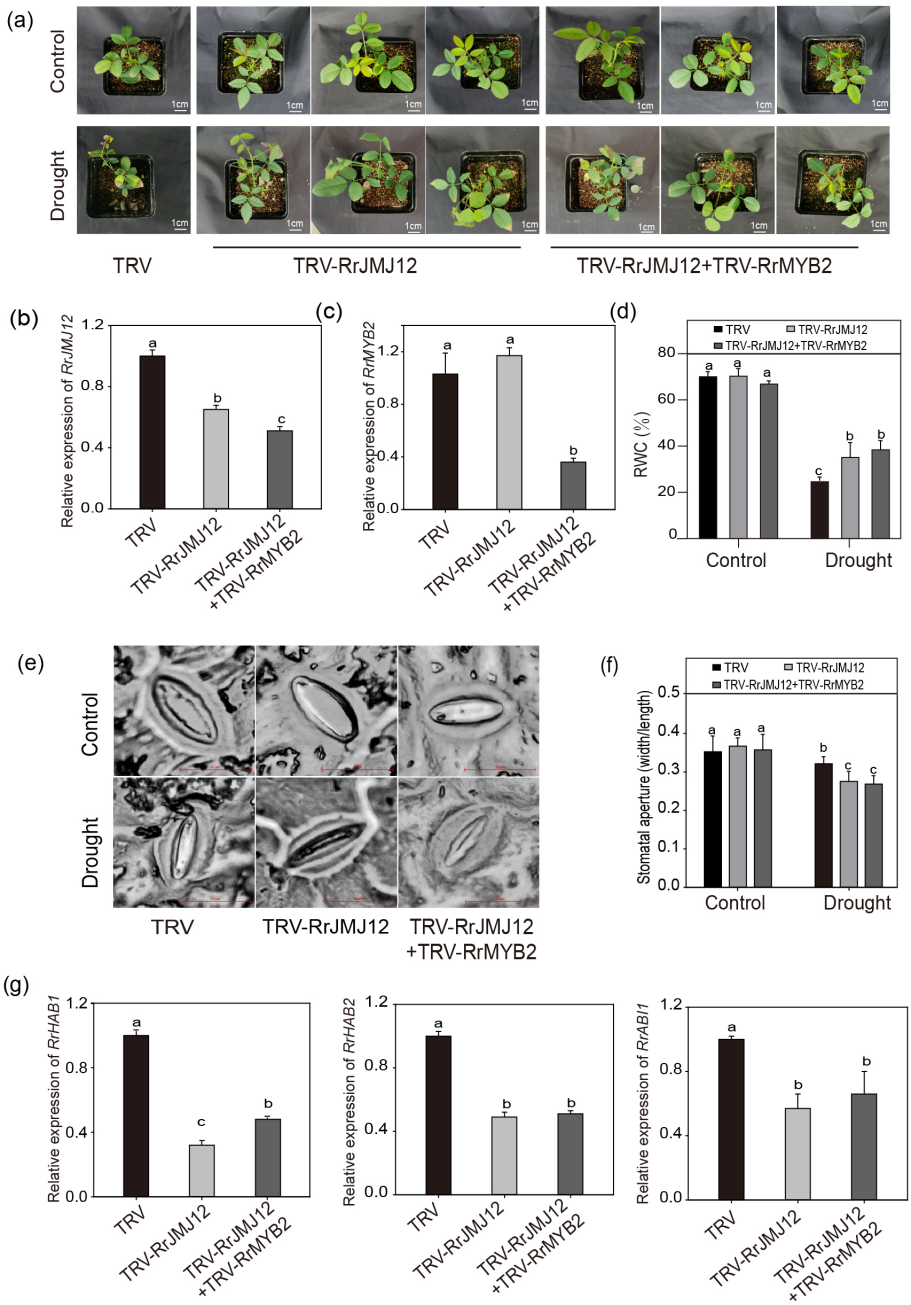
*RrJMJ12* functions independently downstream of *RrMYB2* to negatively regulate the drought resistance. (a) Phenotypes of TRV, TRV‐RrJMJ12 and TRV‐RrJMJ12 + TRV‐RrMYB2 plants before and after drought stress. Bars = 1 cm. (b, c) Expression levels of *RrJMJ12* (b) and *RrMYB2* (c) in the TRV, TRV‐RrJMJ12 and TRV‐RrJMJ12 + TRV‐RrMYB2 rose plants. (d) RWC in the leaves of TRV, TRV‐RrJMJ12 and TRV‐RrJMJ12 + TRV‐RrMYB2 rose plants under control and drought stress conditions. (e), (f) Stomatal phenotypes (e) and aperture size (f) of TRV, TRV‐RrJMJ12 and TRV‐ RrJMJ12 + TRV‐RrMYB2 plants under control and drought stress conditions. Values represent the means ±SD. 10 stomata from three independent experiments. Bars = 10 μm. (g) Expression analysis of *RrHAB1*, *RrHAB2* and *RrABI1* in TRV, TRV‐RrJMJ12 and TRV‐RrJMJ12 + TRV‐RrMYB2 plants. *Rr5.8 s* was used as reference gene. Values represent the means ±SD. Different letters above the bars indicate significant differences determined by one‐way ANOVA analysis followed by Duncan's test (*p* < 0.05).

To further verify the functional relationship between RrMYB2 and RrJMJ12, we silenced *RrMYB2* and *RrJMJ12* simultaneously (Figure 7a–c). As shown in Figure 7d–f, under control conditions, no significant differences in RWC and stomatal aperture were observed between TRV and TRV‐RrJMJ12 + TRV‐RrMYB2 plants. After drought, TRV‐RrJMJ12 + TRV‐RrMYB2 plants showed higher RWC and exhibited smaller stomatal apertures than TRV plants. Moreover, *RrHAB1*, *RrHAB2* and *RrABI1* expression levels were significantly downregulated in TRV‐RrJMJ12 + TRV‐RrMYB2 plants (Figure 7g). These results were consistent with silencing *RrJMJ12* alone, indicating that *RrJMJ12* functions independently downstream of *RrMYB2* in regulating drought responses.

### 3.7 The RrMYB2‐RrJMJ12 Protein Interaction Competitively Reduces the Binding of RrJMJ12 to the CTCTGYTY Motifs

**FIGURE 8:**
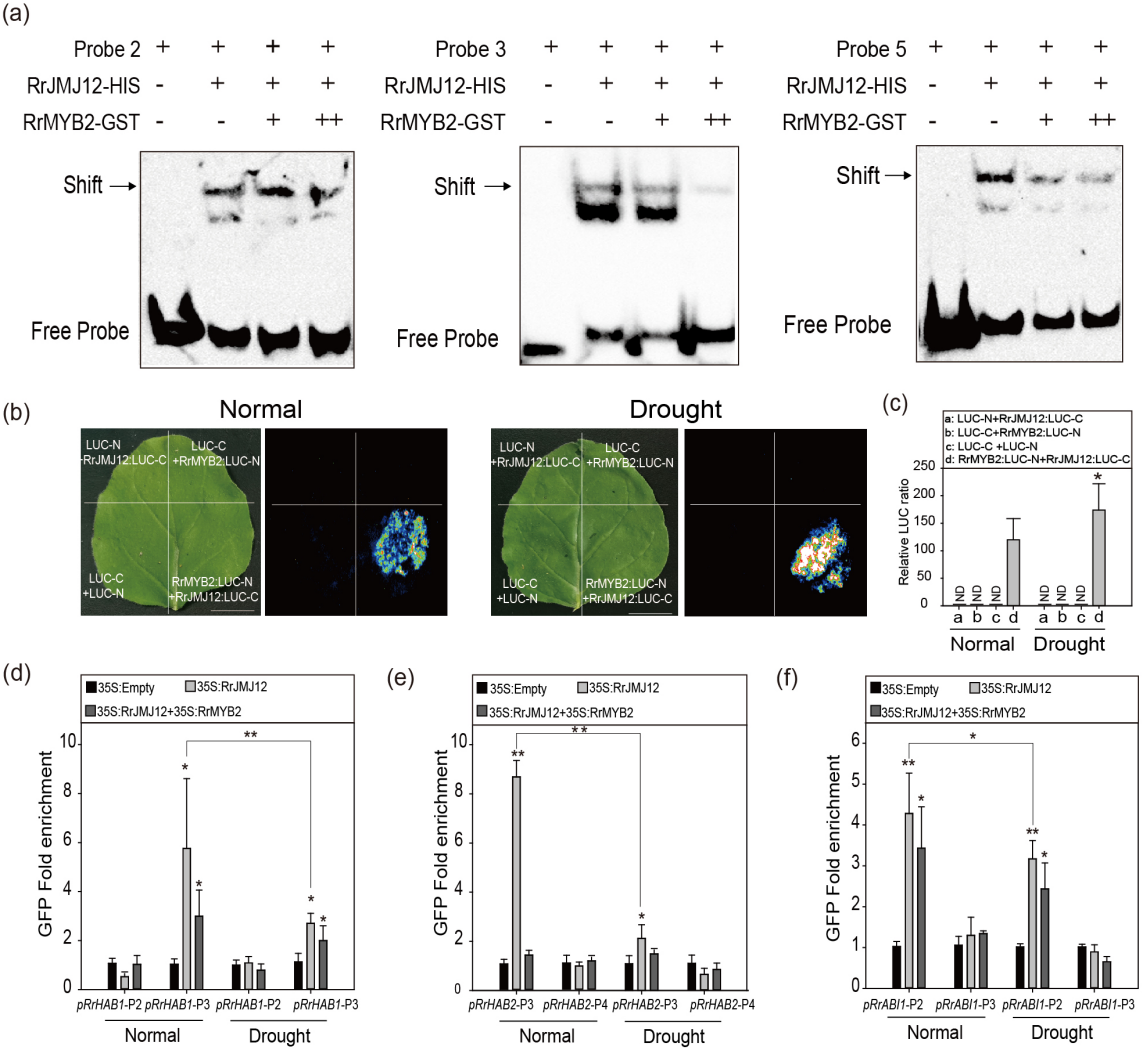
RrMYB2 inhibits the binding of RrJMJ12 to the CTCTGYTY motifs. (a) EMSAs verified that the binding capacity of RrJMJ12 to the CTCTGYTY motifs decreased with the increasing of RrMYB2 proteins. The biotin probes are consistent with that shown in Figure 4. Probes 2, 3 and 5 are respectively located on the *RrHAB1*, *RrHAB2* and *RrABI1* promoters. ‘+’ and ‘++’ represented samples with the addition of one‐ and two‐fold fusion protein, respectively. ‘−’ indicates the absence. The arrow represents the binding band. (b) Split‐LUC complementation assay verified the interaction between RrMYB2 and RrJMJ12 in leaves of *N. benthamiana* under normal and drought conditions. Bars = 2 cm. (c) LUC activity in (b) were calculated using Andor Solis software. Means ± SE were shown from 3 replicates. ND: No Detected. (d–f) ChIP‐qPCR assay of RrJMJ12 binding to the CTCTGYTY motifs in the promoters of *RrHAB1* (d), *RrHAB2* (e) and *RrABI1* (f) in 35S:Empty, or 35S:RrJMJ12, or 35S:RrJMJ12 + 35S:RrMYB2 *R. rugosa* transgenic seedlings under normal and drought conditions. The two regions (with or without CTCTGYTY motifs) selected by each gene are consistent with that shown in Figure 4. Values represent the means ±SD were shown from 3 replicates. Asterisks indicate significant differences determined by Student's t tests (*p < 0.05, **p < 0.01).

Since RrMYB2 interacts with RrJMJ12, we next questioned whether this interaction affects the binding of RrJMJ12 to the CTCTGYTY motifs. We performed EMSAs to examine the binding scenario in detail. As shown in Figure 8a, the binding capacity of RrJMJ12 to the CTCTGYTY motifs substantially decreased with the increasing amounts of RrMYB2 protein, indicating that the interaction between RrMYB2 and RrJMJ12 competitively reduced RrJMJ12 binding to the CTCTGYTY motifs.

To further analyse the effect of RrMYB2 on the RrJMJ12 binding to the CTCTGYTY motifs under drought conditions, we compared the LUC activities under normal and drought conditions. The results showed that the LUC activities were higher under drought conditions than under normal conditions (Figure 8b,c), suggesting that drought stress enhances the interaction between RrMYB2 and RrJMJ12. Then, we transiently infiltrated *R. rugosa* seedlings harbouring 35S:RrJMJ12 or 35S:RrJMJ12 + 35S:RrMYB2 under normal and drought conditions. Overexpression levels of *RrMYB2* and *RrJMJ12* were detected by RT‐qPCR (Figure S9). Subsequently, ChIP‐qPCR was performed on promoter fragments of *RrHAB1*, *RrHAB2* and *RrABI1*, including one containing the CTCTGYTY motifs and one without (as shown in Figure 4g–i). As shown in Figure 8d–f, under drought conditions, RrJMJ12 binding to CTCTGYTY motifs was significantly reduced compared with normal conditions. Furthermore, when co‐expressed with RrMYB2, this binding was further diminished compared to 35S:RrJMJ12 alone.

## 4 Discussion

Although numerous MYB transcription factors involved in abiotic stress responses have been characterised in plants, few have been functionally studied in *R. rugosa* . In this study, we identified RrMYB2, a transcription factor that positively regulates drought resistance. Overexpression of *RrMYB2* significantly improved drought resistance in both rose and *Arabidopsis* (Figure 2 and Figure S2), which was consistent with the results of *MYB2* positively regulating drought resistance in other plants (Abe et al. 2003; Shan et al. 2012).

Under drought conditions, plants alter their physiological morphology and biochemical defences to improve adaptation. Such adaptation includes adjustments of stomatal closure, photosynthetic efficiency, root architecture, leaf morphology and antioxidant clearance (Gill and Tuteja 2010; García‐Caparrós et al. 2021; Ranjan et al. 2022; Luqman et al. 2023). In *RrMYB2* transgenic plants, we observed altered ROS accumulation and peroxidase activity, and enhanced stomatal closure, all of which contributed to improved drought adaptation (Figures 2, 3). Stomata are the main channels for gas exchange between plants and the atmosphere, and they close rapidly under water deficit in response to ABA signalling. In TRV‐RrMYB2 plants, stomatal apertures were significantly larger than in controls (Figure 3d,e). Transcriptome profiling and RT‐qPCR validation revealed that *PP2C* genes *RrHAB1*, *RrHAB2* and *RrABI1* in the ABA signalling pathway were significantly up‐regulated (Figure 3f–h). PP2Cs are pivotal negative regulators in the ABA signalling pathway, with HAB1 and ABI1 specifically characterised as suppressors of drought responses in *Arabidopsis* (Saez et al. 2004, 2006; Park et al. 2009). These results prompted us to investigate how RrMYB2 regulates *PP2C* gene expression.

In general, MYB transcription factors typically activate stress‐responsive genes by binding to MYB‐binding elements in their promoters (Sun et al. 2014; Jian et al. 2022). For instance, Arabidopsis MYB2 activates *RD22* expression by binding to the MYB recognition site (Abe et al. 2003). In our study, although MYB‐binding sites were present in the promoters of *RrHAB1*, *RrHAB2* and *RrABI1* (Figure S5), Y1H assays revealed that RrMYB2 did not bind directly to these promoters (Figure S6a). However, LUC transient transformation assays confirmed that RrMYB2 negatively regulated their expression (Figure S6b–g), suggesting that RrMYB2‐mediated repression requires additional cofactors.

Currently, numerous histone epigenetic modification factors have been reported to play critical roles in plant responses to abiotic stress. For example, the ADA2b‐GCN5 histone acetyltransferase complex interacts with AREB1 to form a ternary protein complex that enhances H3K9ac enrichment at *PtrNAC* gene loci, thereby regulating drought tolerance in poplar (Li, Lin, et al. 2019). In *Arabidopsis*, the RPN1a‐JMJ27 module precisely regulates the dynamic deposition of H3K9me2 at the target gene *RD20* to facilitate drought stress adaptation (Wang et al. 2021). These modifiers are typically recruited to target gene chromatin by forming complexes with other regulatory factors to establish or remove specific epigenetic marks. However, some histone modifiers can independently recognise and modify target genes without relying on other factors. Despite these insights, the mechanisms underlying the specific targeting of most histone‐modifying proteins remain largely unknown. To our knowledge, JMJ12 is the only histone demethylase with a well‐characterised recognition sequence (CTCTGYTY) (Cui et al. 2016), which greatly facilitates the identification of its potential target genes. In this study, we identified the CTCTGYTY motifs in the promoters of *RrHAB1*, *RrHAB2* and *RrABI1* and confirmed that RrJMJ12 binds directly to these sequences using EMSAs. ChIP‐qPCR assays further confirmed that RrJMJ12 regulates these genes by modulating H3K27me3 levels (Figure 4). A series of biochemical analyses revealed that RrMYB2 physically interacts with RrJMJ12 (Figure 5a–c). Furthermore, the repressive effect of RrMYB2 on *RrHAB1*, *RrHAB2* and *RrABI1* was almost eliminated in *RrJMJ12*‐silenced seedlings (Figure 5d–i). These findings indicate that RrMYB2 represses *RrHAB1*, *RrHAB2* and *RrABI1* in an RrJMJ12‐dependent manner.

**FIGURE 9:**
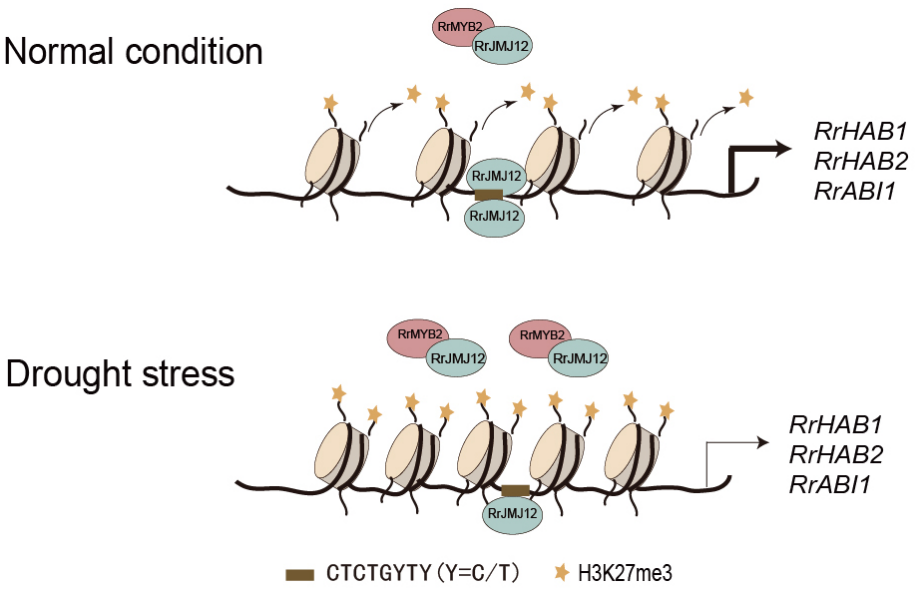
Proposed work model of RrMYB2 regulates drought stress in *R. rugosa* . Under normal conditions, RrJMJ12 regulates the expression of *RrHAB1*, *RrHAB2* and *RrABI1* by binding to the CTCTGYTY motifs, thereby maintaining the normal expression of these genes. Under drought conditions, RrMYB2‐RrJMJ12 protein interaction is enhanced, which competitively reduced RrJMJ12 binding to the CTCTGYTY motifs in the promoters of *RrHAB1*, *RrHAB2* and *RrABI1*, leading to their transcriptional decrease.

JMJ12, the first identified H3K27 histone demethylase in plants, has been implicated in various developmental processes such as flowering, senescence, seed germination and thermomorphogenesis (Noh et al. 2004; Wang, Gao, Gao, Li, et al. 2019; Chen et al. 2020; He et al. 2021; Wang, Gao, Gao, Song, et al. 2019). In this study, genetic evidence demonstrated that RrJMJ12 negatively regulates drought resistance (Figures 6, 7), expanding the functional repertoire of JMJ12. In *Arabidopsis*, JMJ12 cooperates with the transcription factor PIF4 to regulate thermomorphogenesis by removing H3K27me3 from bHLH87, enabling PIF4‐mediated activation. However, whether JMJ12 directly interacts with PIF4 remains unclear (He et al. 2021). In our study, RrMYB2 interacted with RrJMJ12 to form a protein complex, which reduced the binding of RrJMJ12 to the CTCTGYTY motifs in the promoters of *PP2Cs*, thereby reducing their expression. This reduction effect was further enhanced under drought conditions (Figure 8). In conclusion, our study reveals a novel molecular mechanism in which *RrMYB2* confers drought tolerance in *R. rugosa* by repressing *PP2Cs* through cooperation with the histone demethylase RrJMJ12 (Figure 9).

## Author Contributions

Mengjuan Bai and Yating Yang conceived and designed the experiments; Mengjuan Bai, Yating Yang, Yunfeng Gao, Mengmeng Xu, Qianxiang Zhang and Shuo Liu executed parts of the experiments. Jun Lu, Jianwen Wang and Changquan Wang were involved in supervision. Mengjuan Bai and Liguo Feng wrote the manuscript.

## Conflicts of Interest

The authors declare no conflicts of interest.

## Supporting information


**Figures S1‐S9:** pbi70432‐sup‐0001‐FiguresS1‐S9.docx.

**Table S1:** pbi70432‐sup‐0002‐TableS1.xlsx.

**Table S2:** pbi70432‐sup‐0003‐TableS2.xlsx.

**Table S3:** pbi70432‐sup‐0004‐TableS3.xlsx.

**Table S4:** pbi70432‐sup‐0005‐TableS4.xlsx.

**Table S5:** pbi70432‐sup‐0006‐TableS5.xlsx.

## Data Availability

The data that supports the findings of this study is available in the Supporting Information of this article.
