# Supplementary material for: RrMYB2 Regulates Drought Stress via RrJMJ12‐Dependent Epigenetic Modification in Rosa rugosa
Source: Plant Biotechnol J. 2025 Oct 28;24(3):1578–94. doi: 10.1111/pbi.70432 (PMC12946505; doi:10.1111/pbi.70432)
Supplement: Supplementary file 1 — Figures S1‐S9: pbi70432‐sup‐0001‐FiguresS1‐S9.docx. [file PBI-24-1578-s004.docx]

**The following Supporting Information is available for this article:**


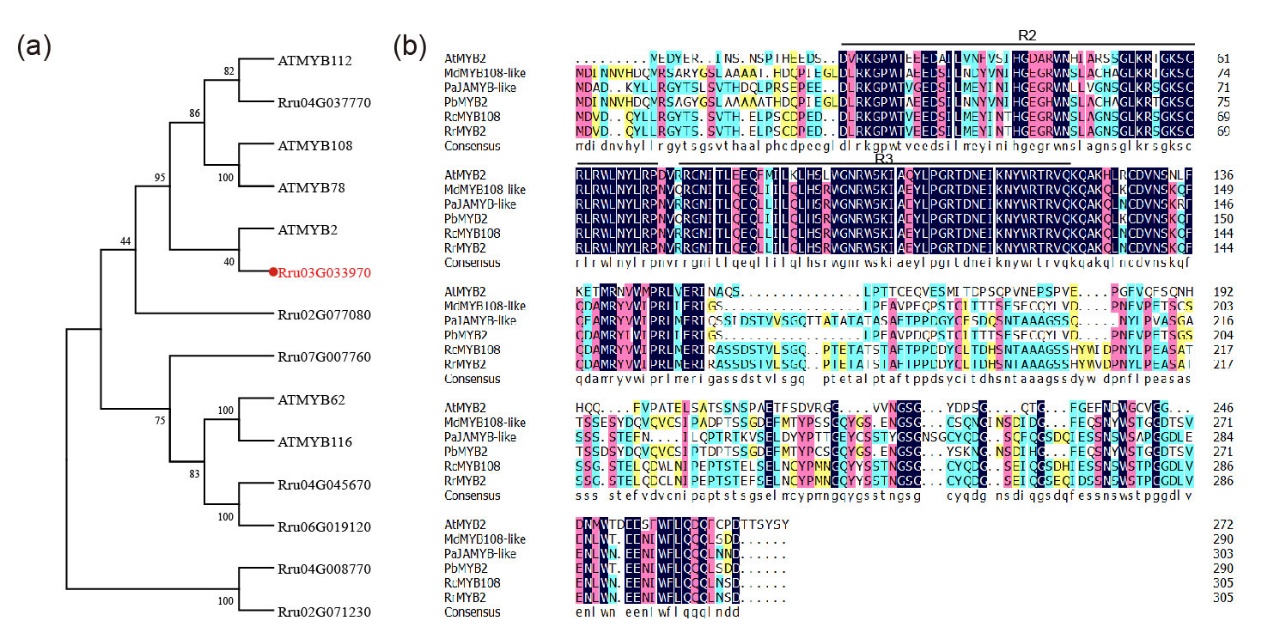


**Figure S1 Identification of *RrMYB2* gene in *R. rugosa.***

(a) Phylogenetic analysis of R2R3-MYB S20 subgroup genes among *Arabidopsis thaliana* and *Rosa rugosa*. Bootstrap values indicate the divergence of each branch. (b) Polypeptide sequence alignment of RrMYB2 and homologous proteins from other species. Black lines indicate R2 and R3 domains.


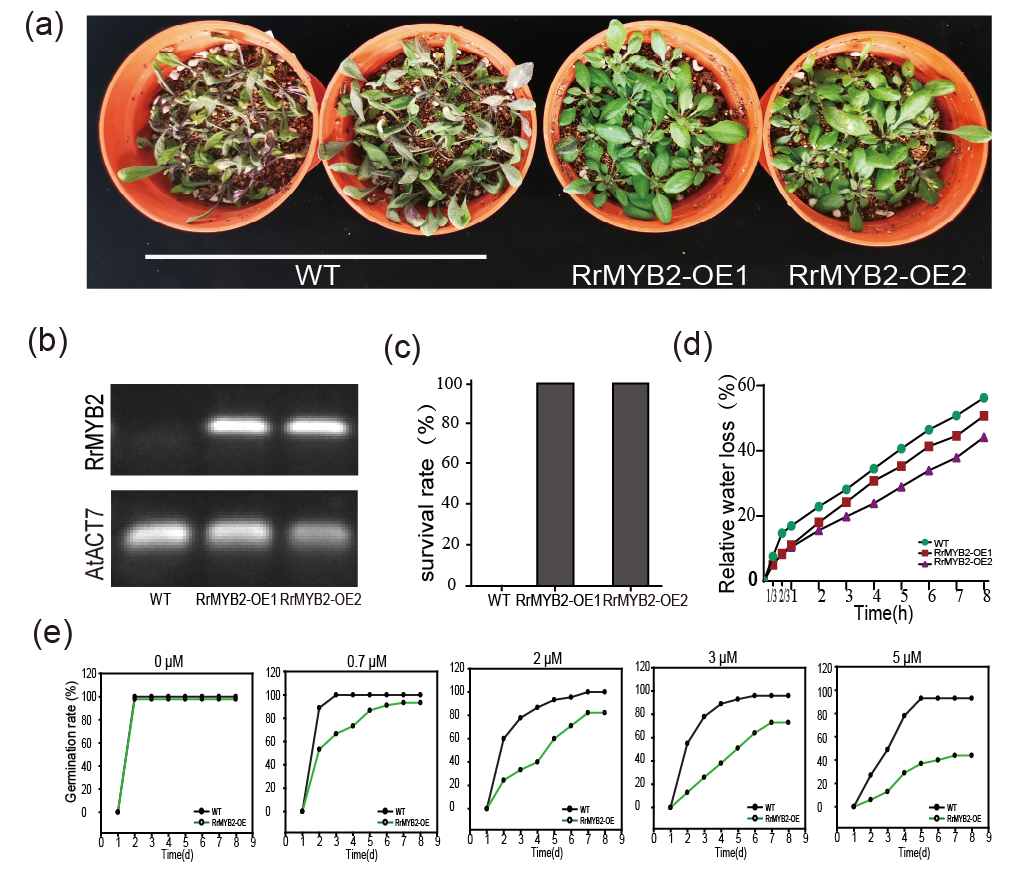


**Figure S2 Ectopic expression of *RrMYB2* enhances drought tolerance in *Arabidopsis*.**

(a) The phenotypes of WT and over-expressing RrMYB2 transgenic lines after drought treatment. (b) Expression levels of *RrMYB2* measured by semi-quantitative RT-PCR in *Arabidopsis*. *AtACT7* was used as reference gene. (c) Survival statistics of WT and RrMYB2-OE lines under drought conditions. (d) Water loss rates of detached leaves from WT and RrMYB2-OE lines under drought conditions. (e) WT and RrMYB2-OE lines seeds were sowed on MS agar plates supplemented with 0, 0.7, 2, 3, 5 μM ABA. The germination rate was counted 10 days after sowing. The germination rate was based on three biological replicates and three technical repeats each with at least 100 seeds (n > 100).


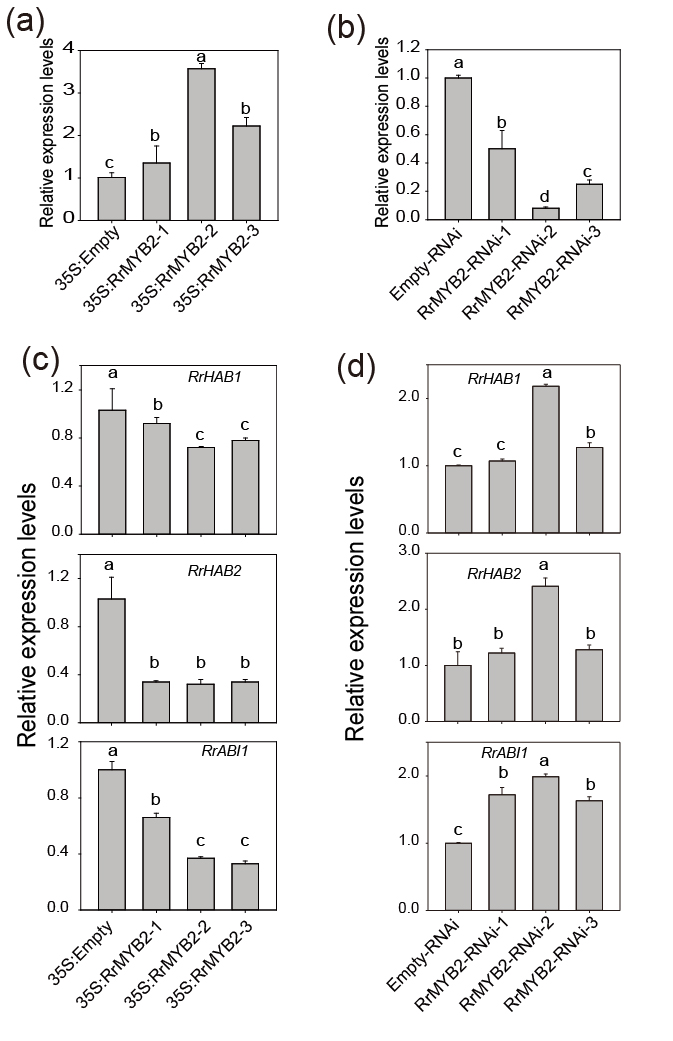


**Figure S3 Expression analysis of *RrMYB2* and *PP2Cs* in the transgenic *R. rugosa* plants.**

(a), (b) Expression levels of *RrMYB2* in the CK (35S:Empty and Empty-RNAi) and transgenic (35S:RrMYB2 and RrMYB2-RNAi) *R. rugosa* roots. *Rr5.8s* was used as reference gene. (c), (d) Expression levels of *PP2Cs* (including *RrHAB1*, *RrHAB2*, and *RrABI1*) in the CK (35S:Empty and Empty-RNAi) and transgenic (35S:RrMYB2 and RrMYB2-RNAi) *R. rugosa* roots. *RrGAPDH* was used as reference gene. Values represent the means ±SD from three replicates. Different letters above the bars indicate significant differences determined by one-way ANOVA analysis followed by Duncan’s test (*P* < 0.05).


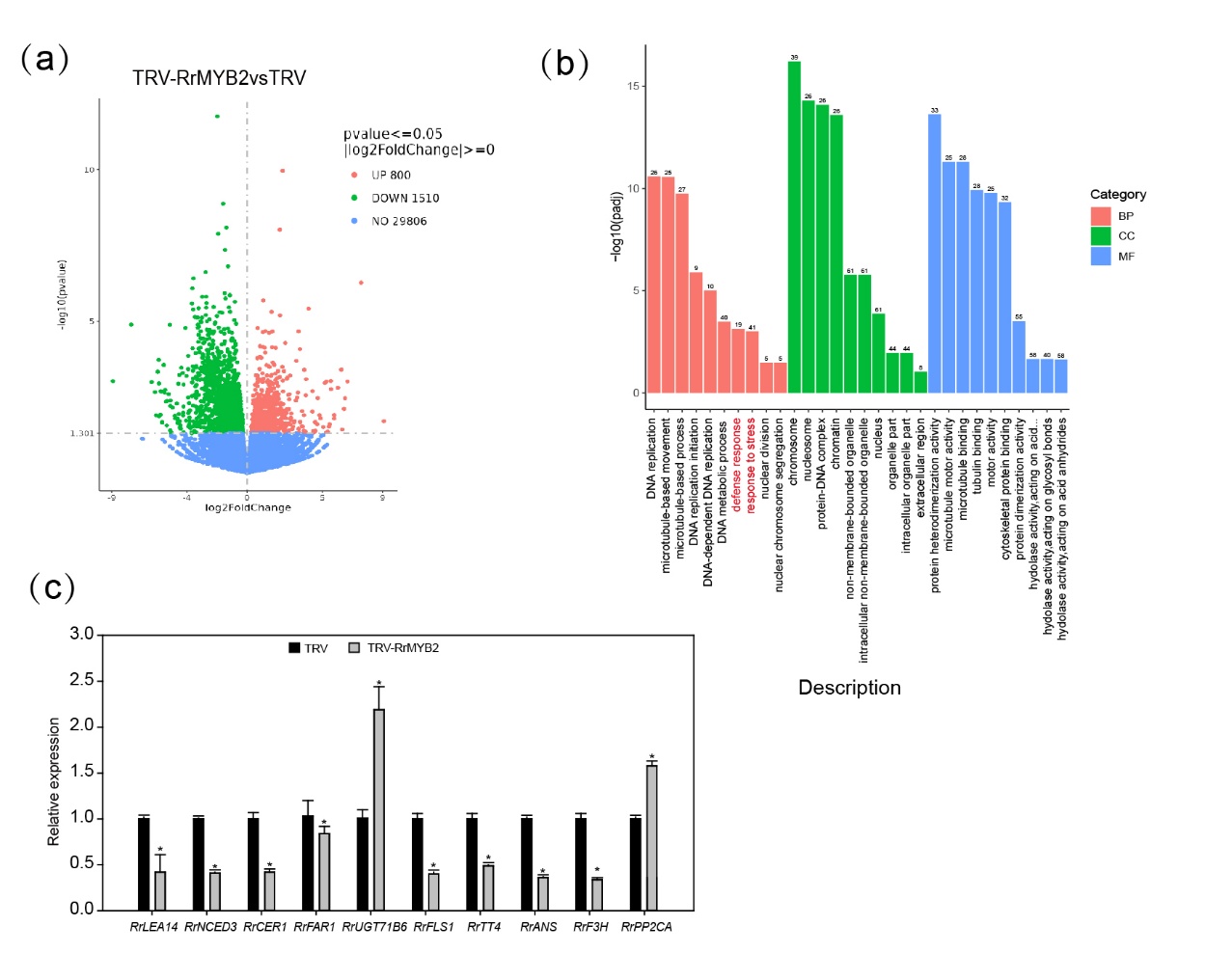


**Figure S4 RNA-seq and stress-related genes expression analysis of TRV and TRV-RrMYB2 plants**

(a) The volcano plot shows the number of DEGs between TRV and TRV-RrMYB2 plants. (b) GO enrichment analysis of DEGs in TRV and TRV-RrMYB2 plants. (c) Expression analysis of ten stress-related genes in TRV and TRV-RrMYB2 plants. *RrGAPDH* was used as reference gene. Values represent the means ±SD from three replicates. Asterisks indicate significant differences determined by Student’s t tests (***P* < 0.01).


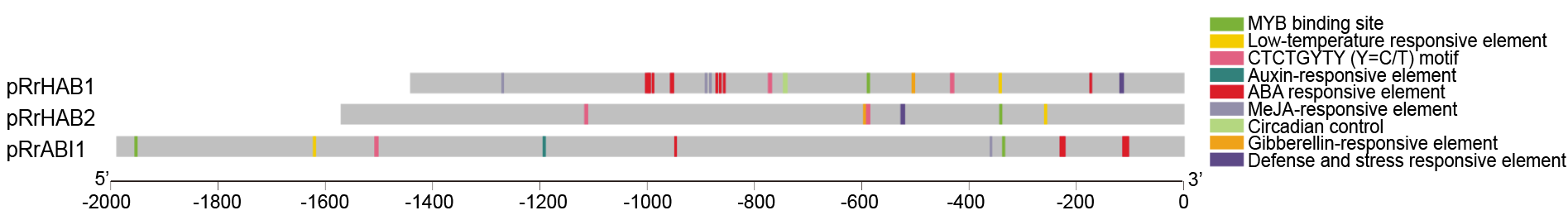


**Figure S5 Analysis of *cis*-elements of three genes promoters.**

The PlantCare online software was used to analyze the *cis*-elements of gene promoter, then visualized via TBtools software, different color blocks represented the positions of elements on the promoter.


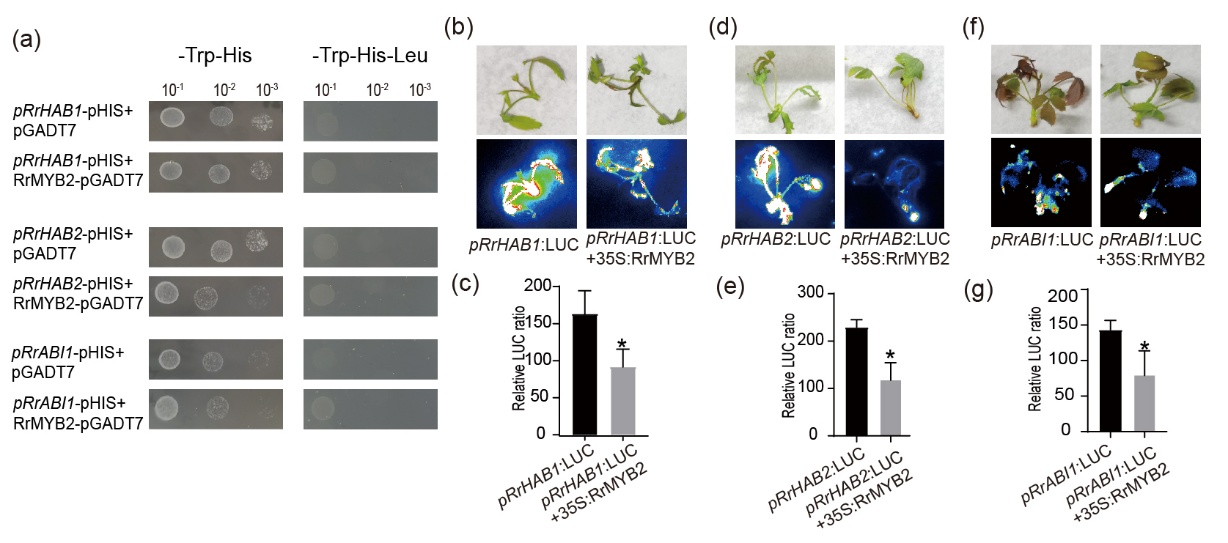


**Figure S6 RrMYB2 indirectly inhibits the expression of *RrHAB1*, *RrHAB2*, and *RrABI1*.**

(a) Y1H assay examining RrMYB2 binding to various gene promoters. Y187 was co-transformed with the indicated combinations of bait and prey vectors. Positive transformants were selected on SD/-Trp/-His, while interactions were tested on SD/-Trp/-His/-Leu. (b), (d), (f) Representative images of LUC transient expression assays in rose seedlings, shown under by bright-field and dark-field conditions. Seedlings were transformed with *pRrHAB1*/*pRrHAB2*/*pRrABI1*:LUC, either alone or co-expressed with 35S:RrMYB2 in a 1:1 ratio. (c), (e), (g) Quantification of LUC activity in (b), (d), (f) using Andor Solis software. Data represent means ± standard error (SE) from approximately 10 replicates. Asterisks indicate significant differences determined by Student’s t tests (**P* < 0.05).


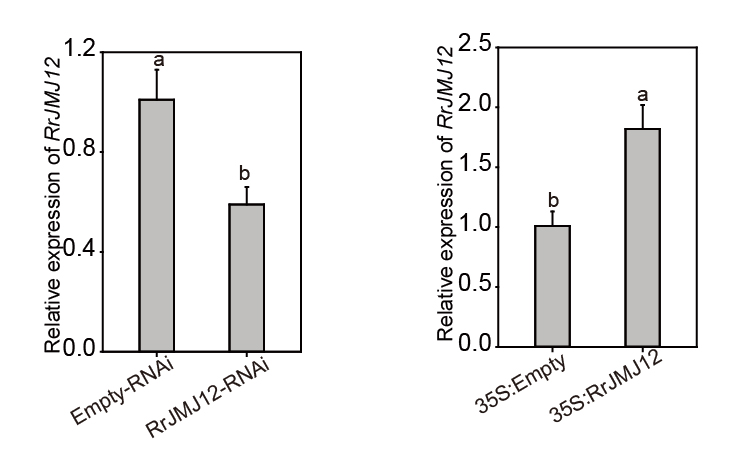


**Figure S7 Expression analysis of *RrJMJ12* in the transiently transgenic *R. rugosa* seedlings.**

Expression levels of *RrJMJ12* in the transgenic *R. rugosa* seedlings harboring Empty-RNAi, RrJMJ12-RNAi, 35S:Empty or 35S:RrJMJ12. *Rr5.8s* was used as reference gene. Values represent the means ±SD from three replicates. Different letters above the bars indicate significant differences determined by one-way ANOVA analysis followed by Duncan’s test (*P* < 0.05).


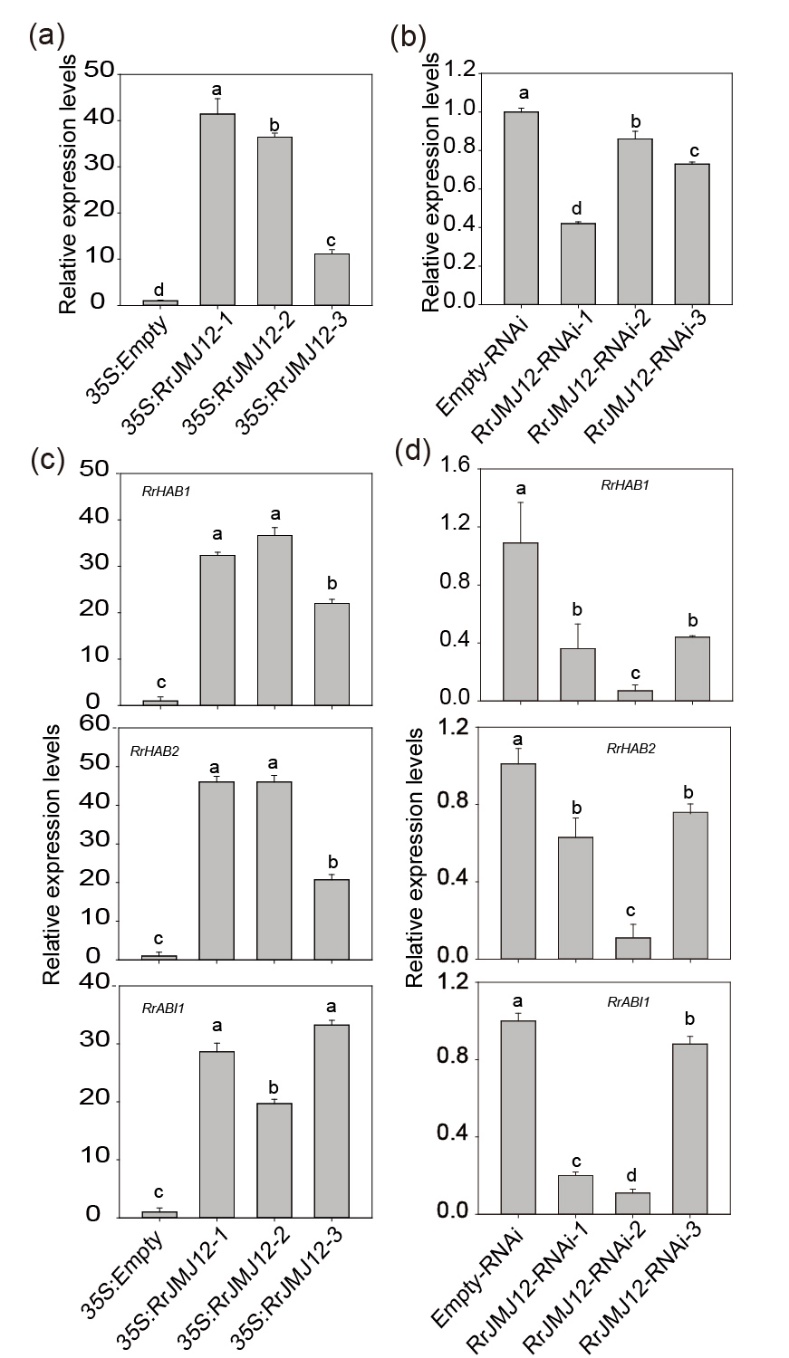


**Figure S8 Expression analysis of *RrJMJ12* and *PP2Cs* in the transgenic *R. rugosa* plants.**

(a), (b) Expression levels of *RrJMJ12* in the CK (35S:Empty and Empty-RNAi) and transgenic (35S: RrJMJ12 and RrJMJ12-RNAi) *R. rugosa* roots. *Rr5.8s* was used as reference gene. (c), (d) Expression levels of *PP2Cs* (including *RrHAB1*, *RrHAB2*, and *RrABI1*) in the CK (35S:Empty and Empty-RNAi) and transgenic (35S:RrJMJ12 and RrJMJ12-RNAi) *R. rugosa* roots. *RrGAPDH* was used as reference gene. Values represent the means ±SD from three replicates. Different letters above the bars indicate significant differences determined by one-way ANOVA analysis followed by Duncan’s test (*P* < 0.05).


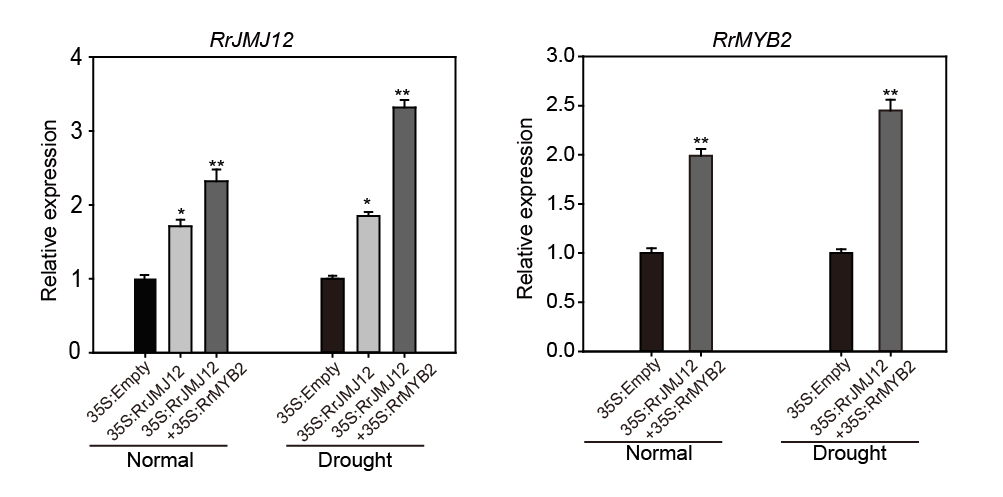


**Figure S9 Expression analysis of *RrJMJ12* and *RrMYB2* in the transiently transgenic *R. rugosa* seedlings.**

Expression levels of *RrJMJ12*, and *RrMYB2* in the transgenic *R. rugosa* seedlings harboring 35S:Empty, 35S:RrJMJ12 and 35S:RrJMJ12+35S:RrMYB2. *Rr5.8s* was used as reference gene. Values represent the means±SD from three replicates. Asterisks indicate significant differences determined by Student’s t tests (**P* < 0.05, ***P* < 0.01).
